# Supplementary material for: Genome-wide association analysis of flowering date in a collection of cultivated olive tree
Source: Hortic Res. 2024 Sep 24;12(1):uhae265. doi: 10.1093/hr/uhae265 (PMC11718396; doi:10.1093/hr/uhae265)
Supplement: Web_Material_uhae265 [file web_material_uhae265.zip › Aqbouch_etal_File_S2.docx]

**Genome-wide association analysis of flowering date in a collection of cultivated olive tree**

Laila Aqbouch^1^, Omar Abou-Saaid^2,3$^, Gautier Sarah^1$^, Lison Zunino^1,4^, Vincent Segura^1^, Pierre Mournet^1,5^, Florelle Bonal^1,5^, Hayat Zaher^3^, Ahmed El Bakkali^6^, Philippe Cubry^4*^, Evelyne Costes^1*¤^, Bouchaib Khadari^1,7*^

1 UMR AGAP Institut, Univ Montpellier, CIRAD, INRAE, Institut Agro, Montpellier, France

2 Université Cadi Ayyad, Laboratoire Biotechnologie et Bio-ingénierie Moléculaire, FST Guéliz, Marrakech, Morocco

3 INRA, UR Amélioration des Plantes, Marrakech, Morocco

4 DIADE, Univ Montpellier, CIRAD, IRD, Montpellier, France

5 CIRAD, UMR AGAP Institut, F-34398 Montpellier, France.

6 INRA, UR Amélioration des Plantes et Conservation des Ressources Phytogénétiques, Meknès, Morocco

7 CBNMed, AGAP Institut, Montpellier, France

$These two authors contributed equally to this work

 *These three last authors contributed equally to this work

 ¤Corresponding author:[evelyne.costes@inrae.fr](mailto:evelyne.costes@inrae.fr)

**S2 File. Supplementary methods**

**Assessment of the reproducibility of capture sequencing experiment**

To assess the reproducibility of the capture sequencing experiment, we replicated three genotypes: *Leccino* (MAR0016), *Picual* (MAR00267), and *Picholine Marocaine* (MAR00540). We calculated the percentage of differences between replicates by dividing the number of different loci by the total number of tested loci, excluding missing alleles. This is done on raw VCF after variant calling (64,835,479 variants) and for the filtered VCF (we reproduced the same VCF filtering pipeline as described before (Table S2), except for the minor allele frequency and non-nuclear data). The error rate of the experiment is approximately 5.96% when using the raw VCF data, but it decreases to 2.5% when using the filtered VCF data (see Table S17).
