# Supplementary material for: Genome-wide association analysis of flowering date in a collection of cultivated olive tree
Source: Hortic Res. 2024 Sep 24;12(1):uhae265. doi: 10.1093/hr/uhae265 (PMC11718396; doi:10.1093/hr/uhae265)
Supplement: Web_Material_uhae265 [file web_material_uhae265.zip › Aqbouch_etal_Table_S1.docx]

| Genotype | Mean depth target sequencing | Mean depth non-captured sequencing | Enrichement rate |
| --- | --- | --- | --- |
| Picholine Marocaine | 49.8 | 1.27 | 39.19 |
| Picholine | 30.6 | 0.77 | 39.66 |
