# Supplementary material for: Genome-wide association analysis of flowering date in a collection of cultivated olive tree
Source: Hortic Res. 2024 Sep 24;12(1):uhae265. doi: 10.1093/hr/uhae265 (PMC11718396; doi:10.1093/hr/uhae265)
Supplement: Web_Material_uhae265 [file web_material_uhae265.zip › Aqbouch_etal_Table_S2.docx]

| Criteria | Number of SNP | Number of samples | Analyses |
| --- | --- | --- | --- |
| Raw data | 64,835,479 | 333 |  |
| Remove 3 Biological replicate-3 duplicated samples-2 full genomes | 64,835,479 | 325 |  |
| Minimum SNP quality of 200 | 58,847,738 | 325 |  |
| Remove indels | 53,404,703 | 325 |  |
| Maximum 3 SNPs within 10 bp | 33,015,216 | 325 |  |
| Minimum mean depth per site 8 and maximum 400 | 967,953 | 325 |  |
| Minimum mean depth per genotype 8 | 967,953 | 325 |  |
| Retain only biallelic SNPs | 869,467 | 325 |  |
| Maximum heterozygosity rate 75% | 860,931 | 325 |  |
| Maximum missing data per SNP 10% | 334,710 | 325 |  |
| Maximum missing data per sample 25% | 334,710 | 318 |  |
| Minor Allele Count 1 | 235,825 | 318 | Genetic structure |
| Minor allele frequency at 5% | 119,614 | 318 |  |
| Nuclear dataset | 119,600 | 318 | Genome-Wide Association Study |
| Imputation + Minor allele frequency at 5% | 118,948 | 318 | Estimation of genomic prediction-based models |
