# Supplementary material for: Genome-wide association analysis of flowering date in a collection of cultivated olive tree
Source: Hortic Res. 2024 Sep 24;12(1):uhae265. doi: 10.1093/hr/uhae265 (PMC11718396; doi:10.1093/hr/uhae265)
Supplement: Web_Material_uhae265 [file web_material_uhae265.zip › Aqbouch_etal_Table_S3.docx]

| Cultivar_name | Code_WOGBM | K1 | K2 | K3 | Genetic group |
| --- | --- | --- | --- | --- | --- |
| Aaleth | 422 | 0.00 | 0.82 | 0.18 | C2 |
| Abbadi Shalal | 613 | 0.94 | 0.06 | 0.00 | C1 |
| Abbadi Abou Gabra-610 | 610 | 1.00 | 0.00 | 0.00 | C1 |
| Abbadi Helo | 656 | 0.96 | 0.04 | 0.00 | C1 |
| Abiad Min Omou | 616 | 0.74 | 0.00 | 0.26 | C1 |
| Abou Anaked | 649 | 0.94 | 0.06 | 0.00 | C1 |
| Abou Monkar | 497 | 0.43 | 0.46 | 0.11 | M |
| Abunara | 79 | 0.55 | 0.00 | 0.45 | M |
| Acebuchera | 215 | 0.03 | 0.00 | 0.97 | C3 |
| Adkam | 611 | 0.96 | 0.04 | 0.00 | C1 |
| Aggezi Shami | 480 | 0.88 | 0.00 | 0.12 | C1 |
| Aggezi Akse | 481 | 0.91 | 0.02 | 0.07 | C1 |
| Aggezi Oshime | 482 | 0.93 | 0.03 | 0.04 | C1 |
| Aglandau | 187 | 0.77 | 0.00 | 0.23 | C1 |
| Agrarez | 436 | 0.80 | 0.00 | 0.20 | C1 |
| Aguenaou | 423 | 0.53 | 0.00 | 0.47 | M |
| Ahia Ousbaa | 438 | 0.39 | 0.55 | 0.06 | M |
| Aîmel | 419 | 0.00 | 1.00 | 0.00 | C2 |
| Aitana | 78 | 0.95 | 0.04 | 0.00 | C1 |
| Akenane | 459 | 0.27 | 0.55 | 0.18 | M |
| Akerma | 418 | 0.75 | 0.00 | 0.25 | C1 |
| Alameno Blanco | 216 | 0.00 | 0.02 | 0.98 | C3 |
| Alameno de Montilla | 218 | 0.00 | 0.38 | 0.62 | M |
| Albatro | 30 | 0.51 | 0.37 | 0.12 | M |
| Allora | 68 | 0.24 | 0.76 | 0.00 | C2 |
| Amargoso | 219 | 0.54 | 0.00 | 0.46 | M |
| Americano | 29 | 0.22 | 0.59 | 0.19 | M |
| Arbequina | 220 | 0.31 | 0.69 | 0.00 | M |
| Ascolana Tenera | 76 | 0.46 | 0.11 | 0.43 | M |
| Azeboudj de Khirane | 445 | 0.32 | 0.65 | 0.03 | M |
| Azeradj | 431 | 0.78 | 0.00 | 0.22 | C1 |
| Azul | 221 | 0.20 | 0.65 | 0.15 | M |
| Baid El Hamam | 491 | 0.87 | 0.02 | 0.10 | C1 |
| Baladi | 487 | 0.93 | 0.02 | 0.06 | C1 |
| Barouni | 410 | 0.04 | 0.00 | 0.96 | C3 |
| Bed Al Iguel | 595 | 0.01 | 0.00 | 0.99 | C3 |
| Beladi | 573 | 1.00 | 0.00 | 0.00 | C1 |
| Beladi-577 | 577 | 1.00 | 0.00 | 0.00 | C1 |
| Beldi | 288 | 0.62 | 0.03 | 0.35 | M |
| Berri Meslal-397 | 397 | 0.00 | 0.79 | 0.21 | C2 |
| Berri Meslal-532 | 532 | 0.00 | 0.80 | 0.20 | C2 |
| Besbessi | 464 | 0.90 | 0.02 | 0.08 | C1 |
| Bez El Anza | 495 | 0.45 | 0.48 | 0.07 | M |
| Biancolilla-83 | 83 | 0.26 | 0.45 | 0.29 | M |
| Bical | 333 | 0.11 | 0.04 | 0.85 | C3 |
| Bissani-578 | 578 | 1.00 | 0.00 | 0.00 | C1 |
| Blanqueta | 222 | 0.23 | 0.77 | 0.00 | C2 |
| Bolvino | 223 | 0.79 | 0.21 | 0.00 | C1 |
| Borriolenca | 334 | 0.30 | 0.56 | 0.14 | M |
| Bosana | 143 | 0.24 | 0.56 | 0.21 | M |
| Bottone di gallo | 84 | 0.08 | 0.61 | 0.31 | M |
| Bouchouika | 394 | 0.03 | 0.00 | 0.97 | C3 |
| Bouchouk Lafayette | 430 | 0.78 | 0.00 | 0.22 | C1 |
| Bouteillan | 189 | 0.73 | 0.00 | 0.27 | C1 |
| Brandofino | 86 | 0.80 | 0.01 | 0.19 | C1 |
| Buga | 398 | 0.37 | 0.22 | 0.41 | M |
| Cairo 7 | 493 | 0.12 | 0.51 | 0.37 | M |
| Calatina | 87 | 0.33 | 0.36 | 0.31 | M |
| Callosina | 391 | 0.02 | 0.14 | 0.84 | C3 |
| Canivano Negro | 224 | 0.00 | 0.13 | 0.87 | C3 |
| Cariasina | 91 | 0.32 | 0.15 | 0.52 | M |
| Carolea | 5 | 0.72 | 0.00 | 0.28 | C1 |
| Carrasqueno de Elvas | 202 | 0.08 | 0.05 | 0.87 | C3 |
| Carrasqueno de Jumilla | 226 | 0.02 | 0.01 | 0.97 | C3 |
| Carrasquillo | 335 | 0.00 | 0.01 | 0.99 | C3 |
| Cassanese | 14 | 0.33 | 0.29 | 0.39 | M |
| Castricianella rapparina | 97 | 0.34 | 0.53 | 0.13 | M |
| Cavalieri | 99 | 0.33 | 0.49 | 0.18 | M |
| Cayon | 191 | 0.72 | 0.00 | 0.28 | C1 |
| Cerasuola | 94 | 0.34 | 0.44 | 0.22 | M |
| Cerezuela | 349 | 0.00 | 0.02 | 0.98 | C3 |
| Chalkidikis | 168 | 0.67 | 0.00 | 0.33 | M |
| Changlot Real | 227 | 0.19 | 0.25 | 0.56 | M |
| Chemchali | 293 | 0.57 | 0.29 | 0.14 | M |
| Chemlal de Kabilye | 420 | 0.00 | 0.78 | 0.22 | C2 |
| Chetoui | 281 | 0.40 | 0.46 | 0.14 | M |
| Chorruo | 229 | 0.02 | 0.00 | 0.98 | C3 |
| Ciciarello | 2 | 0.11 | 0.89 | 0.00 | C2 |
| Cima di Melfi | 92 | 0.24 | 0.67 | 0.09 | M |
| Cirujal | 43 | 0.27 | 0.52 | 0.22 | M |
| Cobrancosa | 203 | 0.07 | 0.02 | 0.91 | C3 |
| Confetto | 89 | 0.74 | 0.00 | 0.26 | C1 |
| Coratina | 101 | 0.13 | 0.71 | 0.16 | C2 |
| Corbella | 230 | 0.49 | 0.40 | 0.11 | M |
| Cordovil de Serpa | 204 | 0.03 | 0.01 | 0.96 | C3 |
| Cornezuelo de Jaen | 231 | 0.45 | 0.00 | 0.55 | M |
| Cornicabra | 232 | 0.03 | 0.02 | 0.95 | C3 |
| Craputea | 88 | 0.28 | 0.53 | 0.19 | M |
| Crastu | 96 | 0.34 | 0.42 | 0.24 | M |
| Crnica-399 | 399 | 0.35 | 0.48 | 0.16 | M |
| Cucca | 32 | 0.33 | 0.47 | 0.20 | M |
| Cuoricino | 35 | 0.36 | 0.64 | 0.00 | M |
| Dhokar | 413 | 0.05 | 0.88 | 0.07 | C2 |
| Djlot Tadmori | 617 | 1.00 | 0.00 | 0.00 | C1 |
| Dolce di Rossano | 11 | 0.42 | 0.58 | 0.00 | M |
| Dressi | 286 | 0.16 | 0.56 | 0.28 | M |
| Dritta di Moscufa | 3 | 0.39 | 0.48 | 0.14 | M |
| Dulzal | 233 | 0.05 | 0.02 | 0.93 | C3 |
| El Salam | 488 | 0.35 | 0.51 | 0.13 | M |
| El Lewa | 494 | 0.91 | 0.03 | 0.06 | C1 |
| Emilia | 36 | 0.45 | 0.55 | 0.00 | M |
| Empeltre | 234 | 0.48 | 0.52 | 0.00 | M |
| Enagua de Arenas | 336 | 0.00 | 0.00 | 1.00 | C3 |
| Ensasi | 599 | 0.99 | 0.01 | 0.00 | C1 |
| Escarabajuelo de Posadas | 235 | 0.00 | 0.01 | 0.99 | C3 |
| Escarabajuelo de Úbeda | 236 | 0.27 | 0.00 | 0.73 | C3 |
| Farga | 338 | 0.46 | 0.54 | 0.00 | M |
| Ferkani | 426 | 0.43 | 0.56 | 0.00 | M |
| Frantoio | 39 | 0.15 | 0.85 | 0.00 | C2 |
| Fulla de Salze | 339 | 0.05 | 0.02 | 0.93 | C3 |
| Galega Vulgar | 205 | 0.00 | 0.49 | 0.51 | M |
| Gemlik | 608 | 0.75 | 0.00 | 0.25 | C1 |
| Gentile di chieti | 15 | 0.40 | 0.60 | 0.00 | M |
| Gerboui | 409 | 0.91 | 0.08 | 0.01 | C1 |
| Gerboui-298 | 298 | 0.47 | 0.40 | 0.14 | M |
| Giarraffa | 105 | 0.53 | 0.00 | 0.47 | M |
| Gordal Sevillana | 108 | 0.53 | 0.00 | 0.47 | M |
| Gordal de Granada | 238 | 0.00 | 0.00 | 1.00 | C3 |
| Grappolo | 41 | 0.29 | 0.59 | 0.12 | M |
| Gremigno di Fauglia | 363 | 0.00 | 1.00 | 0.00 | C2 |
| Grossa di Spagna | 107 | 0.89 | 0.00 | 0.11 | C1 |
| Grossane-194 | 194 | 0.53 | 0.03 | 0.44 | M |
| Habichuelero de Grazalema | 239 | 0.12 | 0.06 | 0.82 | C3 |
| Hamed | 483 | 0.93 | 0.07 | 0.00 | C1 |
| Hamra | 443 | 0.00 | 1.00 | 0.00 | C2 |
| Hemblasi-601 | 601 | 1.00 | 0.00 | 0.00 | C1 |
| Heraktane | 647 | 0.96 | 0.04 | 0.00 | C1 |
| Hojiblanca | 240 | 0.10 | 0.08 | 0.82 | C3 |
| Humaisi | 593 | 1.00 | 0.00 | 0.00 | C1 |
| Idleb | 591 | 0.97 | 0.00 | 0.03 | C1 |
| Ifiri | 457 | 0.29 | 0.47 | 0.24 | M |
| Intosso | 9 | 0.47 | 0.04 | 0.49 | M |
| Istarska crnica | 508 | 0.36 | 0.56 | 0.08 | M |
| Itrana | 17 | 0.42 | 0.19 | 0.39 | M |
| Jabaluna | 241 | 0.27 | 0.00 | 0.73 | C3 |
| Jaropo | 242 | 0.00 | 0.00 | 1.00 | C3 |
| Jlot | 618 | 0.97 | 0.00 | 0.03 | C1 |
| Kaissy | 604 | 1.00 | 0.00 | 0.00 | C1 |
| Kalokerida | 171 | 0.23 | 0.33 | 0.44 | M |
| Karamani | 592 | 0.99 | 0.00 | 0.01 | C1 |
| Karbuncela | 513 | 0.40 | 0.19 | 0.41 | M |
| Karme | 640 | 1.00 | 0.00 | 0.00 | C1 |
| Karolia | 172 | 0.91 | 0.05 | 0.04 | C1 |
| Karydolia | 173 | 0.93 | 0.06 | 0.01 | C1 |
| Kato Drys | 316 | 1.00 | 0.00 | 0.00 | C1 |
| Kerdi | 597 | 1.00 | 0.00 | 0.00 | C1 |
| Khadraya | 449 | 0.17 | 0.68 | 0.16 | M |
| Khalkhali-629 | 629 | 1.00 | 0.00 | 0.00 | C1 |
| Khashabi-631 | 631 | 0.93 | 0.07 | 0.00 | C1 |
| Khnfse | 651 | 1.00 | 0.00 | 0.00 | C1 |
| Khodieri | 627 | 1.00 | 0.00 | 0.00 | C1 |
| Kolybada | 174 | 0.81 | 0.08 | 0.11 | C1 |
| Kossiem | 496 | 0.16 | 0.44 | 0.40 | M |
| Kothreiki | 176 | 0.82 | 0.18 | 0.00 | C1 |
| Koutsourelia | 177 | 0.44 | 0.56 | 0.00 | M |
| Lastovka | 506 | 0.50 | 0.50 | 0.00 | M |
| Lastrino | 23 | 0.00 | 0.77 | 0.23 | C2 |
| Lazzero di prata | 367 | 0.19 | 0.39 | 0.42 | M |
| Lazzero | 25 | 0.00 | 0.96 | 0.04 | C2 |
| Leccino | 16 | 0.20 | 0.58 | 0.22 | M |
| Leccio Maremmano | 112 | 0.36 | 0.64 | 0.00 | M |
| Lechin de Sevilla | 243 | 0.00 | 0.41 | 0.59 | M |
| Lechin de Granada | 340 | 0.00 | 0.37 | 0.63 | M |
| Lentisca-206 | 206 | 0.00 | 0.50 | 0.50 | M |
| Lentisca-244 | 244 | 0.07 | 0.34 | 0.59 | M |
| Limoncillo | 341 | 0.02 | 0.05 | 0.93 | C3 |
| Lloron de Atarfe | 245 | 0.07 | 0.00 | 0.93 | C3 |
| Llumeta | 343 | 0.33 | 0.67 | 0.00 | M |
| Loaime | 344 | 0.01 | 0.00 | 0.99 | C3 |
| Lucques | 195 | 0.64 | 0.16 | 0.19 | M |
| Lumbardeska | 500 | 0.43 | 0.17 | 0.40 | M |
| Lumiaro | 113 | 0.71 | 0.00 | 0.29 | C1 |
| Machorron | 247 | 0.02 | 0.03 | 0.95 | C3 |
| Madonna dell impruneta | 50 | 0.35 | 0.61 | 0.05 | M |
| Madural-208 | 208 | 0.01 | 0.03 | 0.96 | C3 |
| Mahati-615 | 615 | 0.94 | 0.06 | 0.00 | C1 |
| Maiatica di Ferrandina | 4 | 0.80 | 0.20 | 0.00 | C1 |
| Mancanilha Algarvia | 207 | 0.55 | 0.00 | 0.45 | M |
| Manzanilla Cacerena | 248 | 0.06 | 0.03 | 0.91 | C3 |
| Manzanilla de Sevilla | 251 | 0.02 | 0.01 | 0.97 | C3 |
| Manzanilla de Agua | 345 | 0.00 | 0.00 | 1.00 | C3 |
| Manzanilla de Hellin | 346 | 0.04 | 0.01 | 0.95 | C3 |
| Manzanilla de Montefrio | 250 | 0.01 | 0.00 | 0.99 | C3 |
| Maraki | 485 | 0.79 | 0.14 | 0.07 | C1 |
| Maremmano | 368 | 0.31 | 0.63 | 0.05 | M |
| Masabi | 585 | 0.76 | 0.00 | 0.24 | C1 |
| Mastoidis | 178 | 0.39 | 0.61 | 0.00 | M |
| Maurino | 118 | 0.30 | 0.56 | 0.14 | M |
| Mavreya | 179 | 0.31 | 0.69 | 0.00 | M |
| Mawi | 620 | 0.93 | 0.07 | 0.00 | C1 |
| Meloky | 490 | 0.87 | 0.10 | 0.03 | C1 |
| Meslala | 535 | 0.09 | 0.00 | 0.91 | C3 |
| Mesyaf-641 | 641 | 0.93 | 0.02 | 0.05 | C1 |
| Mesyaf-662 | 662 | 0.98 | 0.02 | 0.00 | C1 |
| Mignolo Cerretano | 46 | 0.16 | 0.61 | 0.23 | M |
| Mignolo | 49 | 0.01 | 0.99 | 0.00 | C2 |
| Minekiri | 634 | 1.00 | 0.00 | 0.00 | C1 |
| Mohazam Abou Satl | 614 | 0.95 | 0.05 | 0.00 | C1 |
| Mollar de Cieza | 348 | 0.04 | 0.00 | 0.96 | C3 |
| Moraiolo | 119 | 0.23 | 0.62 | 0.15 | M |
| Morchiaio | 117 | 0.38 | 0.61 | 0.01 | M |
| Morchione | 67 | 0.38 | 0.49 | 0.13 | M |
| Morcone | 45 | 0.11 | 0.82 | 0.06 | C2 |
| Morello a punta | 372 | 0.30 | 0.57 | 0.14 | M |
| Moresca | 121 | 0.76 | 0.00 | 0.24 | C1 |
| Morisca | 254 | 0.01 | 0.01 | 0.98 | C3 |
| Morona | 246 | 0.02 | 0.00 | 0.97 | C3 |
| Morrut | 350 | 0.49 | 0.35 | 0.16 | M |
| Mortellino | 373 | 0.00 | 0.97 | 0.03 | C2 |
| Nasitana Frutto Grosso | 128 | 0.55 | 0.33 | 0.12 | M |
| Neb Jmel-283 | 283 | 0.45 | 0.41 | 0.14 | M |
| Negral de Sabinan-255 | 255 | 0.78 | 0.09 | 0.13 | C1 |
| Negrillo de Arjona | 256 | 0.04 | 0.03 | 0.93 | C3 |
| Negrillo de Estepa | 351 | 0.00 | 0.06 | 0.94 | C3 |
| Negrillo de Iznalloz | 352 | 0.00 | 0.00 | 1.00 | C3 |
| Negrillo Redondo | 257 | 0.02 | 0.02 | 0.95 | C3 |
| Negrita | 210 | 0.14 | 0.00 | 0.86 | C3 |
| Nerba | 123 | 0.78 | 0.00 | 0.22 | C1 |
| Nevado Azul | 354 | 0.00 | 0.00 | 1.00 | C3 |
| Nevado Basto | 225 | 0.00 | 0.00 | 1.00 | C3 |
| Nevado Rizado | 355 | 0.00 | 0.05 | 0.95 | C3 |
| Nocellara del Belice | 129 | 0.80 | 0.00 | 0.20 | C1 |
| Nocellara Etnea | 126 | 0.64 | 0.00 | 0.36 | M |
| Nociara | 8 | 0.13 | 0.64 | 0.23 | M |
| Ocal | 258 | 0.11 | 0.00 | 0.89 | C3 |
| Ogliarola del Bradano | 134 | 0.05 | 0.92 | 0.04 | C2 |
| Ogliarola del Vulture | 133 | 0.45 | 0.55 | 0.00 | M |
| Ojo de Liebre | 259 | 0.07 | 0.01 | 0.93 | C3 |
| Olivastra di Montalcino | 375 | 0.00 | 0.99 | 0.01 | C2 |
| Olivastra di Populonia | 72 | 0.01 | 0.93 | 0.07 | C2 |
| Olivo de Mancha Real | 260 | 0.00 | 0.00 | 1.00 | C3 |
| Olivo del Mulino | 165 | 0.36 | 0.19 | 0.45 | M |
| Olivo di San Lorenzo | 69 | 0.29 | 0.57 | 0.14 | M |
| Olivo di Casavecchia | 34 | 0.19 | 0.45 | 0.36 | M |
| Olivo di Mandanici | 136 | 0.05 | 0.95 | 0.00 | C2 |
| Ornellaia | 73 | 0.33 | 0.50 | 0.17 | M |
| Ottobratica | 1 | 0.08 | 0.92 | 0.00 | C2 |
| Palomar | 262 | 0.45 | 0.44 | 0.11 | M |
| Patronet | 263 | 0.50 | 0.33 | 0.18 | M |
| Pendolino-162 | 162 | 0.18 | 0.65 | 0.17 | M |
| Piangente | 58 | 0.18 | 0.68 | 0.14 | M |
| Picholine | 196 | 0.52 | 0.17 | 0.31 | M |
| Picholine Marocaine | 540 | 0.00 | 0.01 | 0.99 | C3 |
| Pico Limon de Grazalema | 265 | 0.06 | 0.01 | 0.93 | C3 |
| Picual | 267 | 0.00 | 0.05 | 0.95 | C3 |
| Picudo | 356 | 0.00 | 0.00 | 1.00 | C3 |
| Pidicuddara | 157 | 0.38 | 0.41 | 0.21 | M |
| Pikrolia | 180 | 0.29 | 0.60 | 0.12 | M |
| Plementa Bjelica | 402 | 0.50 | 0.32 | 0.18 | M |
| Puntoza | 499 | 0.36 | 0.17 | 0.47 | M |
| Rachati-181 | 181 | 0.46 | 0.52 | 0.02 | M |
| Racimal | 268 | 0.02 | 0.00 | 0.98 | C3 |
| Rapasayo | 357 | 0.00 | 0.28 | 0.72 | C3 |
| Ravece | 140 | 0.06 | 0.00 | 0.94 | C3 |
| Razzaio | 63 | 0.38 | 0.61 | 0.02 | M |
| Rechino | 269 | 0.03 | 0.05 | 0.92 | C3 |
| Redondal | 211 | 0.01 | 0.02 | 0.96 | C3 |
| Remmani | 588 | 1.00 | 0.00 | 0.00 | C1 |
| Ronde de la Menara | 543 | 0.54 | 0.00 | 0.46 | M |
| Ronde de Miliana | 428 | 0.00 | 0.02 | 0.98 | C3 |
| Rossellino Cerretano | 379 | 0.30 | 0.59 | 0.11 | M |
| Rossellino | 60 | 0.16 | 0.78 | 0.06 | C2 |
| Rossello | 62 | 0.31 | 0.63 | 0.06 | M |
| Rougette de Mitidja | 444 | 0.40 | 0.39 | 0.21 | M |
| Royal de Cazorla | 270 | 0.10 | 0.02 | 0.88 | C3 |
| Sabatera | 271 | 0.42 | 0.46 | 0.12 | M |
| Salicino | 71 | 0.32 | 0.36 | 0.31 | M |
| Salonenque | 197 | 0.91 | 0.06 | 0.03 | C1 |
| Samo | 404 | 0.39 | 0.16 | 0.45 | M |
| San Francesco | 64 | 0.48 | 0.46 | 0.06 | M |
| Sant Agostino | 146 | 0.72 | 0.00 | 0.27 | C1 |
| Santa Martinenga | 145 | 0.44 | 0.29 | 0.27 | M |
| Sayali | 287 | 0.26 | 0.36 | 0.37 | M |
| Sebhawy | 492 | 0.00 | 0.01 | 0.99 | C3 |
| Sevillano de Jumilla | 272 | 0.06 | 0.00 | 0.94 | C3 |
| Sevillenca | 358 | 0.40 | 0.30 | 0.30 | M |
| Simjaca | 502 | 0.46 | 0.13 | 0.41 | M |
| Sinopolese | 18 | 0.38 | 0.50 | 0.12 | M |
| Sivigliana da Olio | 150 | 0.00 | 0.81 | 0.19 | C2 |
| Souidi | 458 | 0.05 | 0.95 | 0.00 | C2 |
| Storta | 406 | 0.45 | 0.30 | 0.25 | M |
| Sukkare | 663 | 0.95 | 0.05 | 0.00 | C1 |
| Tabelout | 437 | 0.07 | 0.93 | 0.00 | C2 |
| Tarabelsi | 632 | 1.00 | 0.00 | 0.00 | C1 |
| Tebabs | 661 | 0.92 | 0.08 | 0.00 | C1 |
| Teffah | 427 | 0.61 | 0.01 | 0.39 | M |
| Tempranillo de Yeste-274 | 274 | 0.09 | 0.00 | 0.91 | C3 |
| Toffahi-486 | 486 | 0.91 | 0.00 | 0.09 | C1 |
| Toffahi-621 | 621 | 1.00 | 0.00 | 0.00 | C1 |
| Tonda Iblea | 12 | 0.70 | 0.00 | 0.30 | M |
| Tounsi-461 | 461 | 0.62 | 0.28 | 0.11 | M |
| Trillo | 382 | 0.00 | 0.62 | 0.38 | M |
| Unkown-OT2-537 | 537 | 0.33 | 0.67 | 0.00 | M |
| Unkown-OZ1-538 | 538 | 0.00 | 0.74 | 0.26 | C2 |
| Unkown-VS1-544 | 544 | 0.00 | 0.73 | 0.27 | C2 |
| Unkown-VS2-545 | 545 | 0.00 | 0.75 | 0.25 | C2 |
| Unkown-VS5-547 | 547 | 0.00 | 0.75 | 0.25 | C2 |
| Uovo di Piccione | 141 | 0.74 | 0.00 | 0.26 | C1 |
| Vaddarica | 164 | 0.30 | 0.57 | 0.13 | M |
| Varudo | 273 | 0.03 | 0.00 | 0.97 | C3 |
| Varudo-275 | 275 | 0.04 | 0.00 | 0.96 | C3 |
| Vasilikada | 186 | 0.40 | 0.32 | 0.28 | M |
| Velika Lastovka | 515 | 0.42 | 0.12 | 0.46 | M |
| Vera | 276 | 0.39 | 0.61 | 0.00 | M |
| Verdala | 278 | 0.01 | 0.00 | 0.99 | C3 |
| Verdale | 199 | 0.52 | 0.00 | 0.47 | M |
| Verdial de Badajoz | 342 | 0.00 | 0.00 | 1.00 | C3 |
| Verdial de Huevar | 213 | 0.01 | 0.08 | 0.92 | C3 |
| Verdial transmontana | 214 | 0.11 | 0.00 | 0.89 | C3 |
| Verdiell | 279 | 0.28 | 0.51 | 0.21 | M |
| Villalonga | 201 | 0.57 | 0.23 | 0.19 | M |
| Wateken | 484 | 0.87 | 0.08 | 0.05 | C1 |
| Zael Al Muhra | 639 | 1.00 | 0.00 | 0.00 | C1 |
| Zaity | 603 | 1.00 | 0.00 | 0.00 | C1 |
| Zalmati-299 | 299 | 0.06 | 0.84 | 0.10 | C2 |
| Zarza | 280 | 0.00 | 0.41 | 0.59 | M |
| Zeboudj boudoudane | 441 | 0.33 | 0.45 | 0.23 | M |
| Zeletni | 421 | 0.00 | 0.85 | 0.15 | C2 |
