# Supplementary material for: Genome-wide association analysis of flowering date in a collection of cultivated olive tree
Source: Hortic Res. 2024 Sep 24;12(1):uhae265. doi: 10.1093/hr/uhae265 (PMC11718396; doi:10.1093/hr/uhae265)
Supplement: Web_Material_uhae265 [file web_material_uhae265.zip › Aqbouch_etal_Table_S4.docx]

| **Geographical_region** | **Countries** | **C1** | **C2** | **C3** | **M** |
| --- | --- | --- | --- | --- | --- |
| **Western** | Morocco |  | 18% | 6% | 1% |
|  | Portugal |  |  | 11% | 3% |
|  | Spain | 3% | 3% | 76% | 16% |
|  | **Total** | **3%** | **21%** | **93%** | **21%** |
| **Central** | Algeria | 5% | 21% | 1% | 7% |
|  | Tunisia | 3% | 6% | 1% | 7% |
|  | France | 5% |  |  | 3% |
|  | Italy | 15% | 52% | 1% | 44% |
|  | Slovenia |  |  |  | 4% |
|  | Croatia |  |  |  | 5% |
|  | **Total** | **28%** | **79%** | **4%** | **70%** |
| **Eastern** | Greece | 5% |  |  | 6% |
|  | Egypt | 14% |  | 1% | 4% |
|  | Cyprus | 1% |  |  |  |
|  | Lebanon | 6% |  |  |  |
|  | Syria | 43% |  | 1% |  |
|  | **total** | **70%** | **0%** | **3%** | **10%** |
| **Number of genotypes** |  | 79 | 33 | 71 | 135 |
