# Supplementary material for: Genome-wide association analysis of flowering date in a collection of cultivated olive tree
Source: Hortic Res. 2024 Sep 24;12(1):uhae265. doi: 10.1093/hr/uhae265 (PMC11718396; doi:10.1093/hr/uhae265)
Supplement: Web_Material_uhae265 [file web_material_uhae265.zip › Aqbouch_etal_Table_S5.docx]

|  | C1 | C2 | C3 |
| --- | --- | --- | --- |
| mean | 116.53 | 117.55 | 115.47 |
| C1 |  | 0.0066** | 5.5e-06*** |
| C2 |  |  | 2.9e-08*** |
