# Supplementary material for: Genome-wide association analysis of flowering date in a collection of cultivated olive tree
Source: Hortic Res. 2024 Sep 24;12(1):uhae265. doi: 10.1093/hr/uhae265 (PMC11718396; doi:10.1093/hr/uhae265)
Supplement: Web_Material_uhae265 [file web_material_uhae265.zip › Aqbouch_etal_Table_S6.docx]

| **Model** | **Model equation** | **Kinship method** | **AIC** | **BIC** |
| --- | --- | --- | --- | --- |
| Q model | BLUP_FFD = Genotype + Structure |  | 864.5273 | 872.0514 |
| u model | BLUP_FFD = Genotype + \|u | Weir&Goudet | **564.2286** | **567.9907** |
|  |  | vanRaden | **558.3438** | **562.1059** |
| u+Q model | BLUP_FFD = Genotype + \|u+ Structure | Weir&Goudet | 565.9953 | 577.2814 |
|  |  | vanRaden | 560.4763 | 571.7625 |
