# Supplementary material for: Genome-wide association analysis of flowering date in a collection of cultivated olive tree
Source: Hortic Res. 2024 Sep 24;12(1):uhae265. doi: 10.1093/hr/uhae265 (PMC11718396; doi:10.1093/hr/uhae265)
Supplement: Web_Material_uhae265 [file web_material_uhae265.zip › Aqbouch_etal_Table_S7.docx]

| Cultivar_Name | Code_WOGBM | BLUP_FFD |
| --- | --- | --- |
| Ogliarola del Bradano | 134 | 121.1 |
| Allora | 68 | 120.9 |
| Lazzero di prata | 367 | 120.7 |
| Madonna dell impruneta | 50 | 120.6 |
| Grappolo | 41 | 120.4 |
| Emilia | 36 | 120.3 |
| Ogliarola del Vulture | 133 | 120.1 |
| Olivastra di Populonia | 72 | 120.1 |
| Lastrino | 23 | 119.9 |
| Karme | 640 | 119.6 |
| Gremigno di Fauglia | 363 | 119.5 |
| Morcone | 45 | 119.4 |
| Abbadi Helo | 656 | 119.4 |
| Istarska crnica | 508 | 119.4 |
| Zeboudj boudoudane | 441 | 119.3 |
| Dolce di Rossano | 11 | 119.3 |
| Americano | 29 | 119.3 |
| Minekiri | 634 | 119.2 |
| Dritta di Moscufa | 3 | 119.2 |
| Moraiolo | 119 | 119.1 |
| Morello a punta | 372 | 119.1 |
| Leccino | 16 | 119.1 |
| Negrillo de Arjona | 256 | 119.0 |
| Rossellino Cerretano | 379 | 119.0 |
| Olivo di Casavecchia | 34 | 119.0 |
| Piangente | 58 | 118.9 |
| Olivo di San Lorenzo | 69 | 118.9 |
| Leccio Maremmano | 112 | 118.9 |
| Abbadi Shalal | 613 | 118.9 |
| Nociara | 8 | 118.9 |
| Blanqueta | 222 | 118.8 |
| Sinopolese | 18 | 118.7 |
| Pendolino-162 | 162 | 118.6 |
| Ottobratica | 1 | 118.6 |
| Plementa Bjelica | 402 | 118.6 |
| Adkam | 611 | 118.6 |
| Meloky | 490 | 118.6 |
| Morchiaio | 117 | 118.5 |
| Sivigliana da Olio | 150 | 118.5 |
| Cerezuela | 349 | 118.5 |
| Mahati-615 | 615 | 118.3 |
| Heraktane | 647 | 118.3 |
| Maurino | 118 | 118.3 |
| Razzaio | 63 | 118.3 |
| Remmani | 588 | 118.3 |
| Ahia Ousbaa | 438 | 118.3 |
| Dhokar | 413 | 118.2 |
| Karbuncela | 513 | 118.2 |
| Mesyaf-641 | 641 | 118.2 |
| Gentile di chieti | 15 | 118.2 |
| Khalkhali-629 | 629 | 118.1 |
| Karamani | 592 | 118.1 |
| Pikrolia | 180 | 118.1 |
| Hamed | 483 | 118.1 |
| Picual | 267 | 118.0 |
| Rachati-181 | 181 | 118.0 |
| Maremmano | 368 | 118.0 |
| Tebabs | 661 | 118.0 |
| Mawi | 620 | 117.9 |
| Zael Al Muhra | 639 | 117.9 |
| Picudo | 356 | 117.9 |
| Itrana | 17 | 117.8 |
| Santa Martinenga | 145 | 117.8 |
| Bissani-578 | 578 | 117.8 |
| Abbadi Abou Gabra-610 | 610 | 117.8 |
| Abou Anaked | 649 | 117.8 |
| Mesyaf-662 | 662 | 117.8 |
| Lentisca-206 | 206 | 117.8 |
| Tounsi-461 | 461 | 117.8 |
| Hamra | 443 | 117.7 |
| Ascolana Tenera | 76 | 117.7 |
| Frantoio | 39 | 117.7 |
| Calatina | 87 | 117.7 |
| Lumbardeska | 500 | 117.7 |
| Ferkani | 426 | 117.6 |
| Lentisca-244 | 244 | 117.6 |
| Samo | 404 | 117.6 |
| Morchione | 67 | 117.6 |
| Khadraya | 449 | 117.6 |
| Olivo di Mandanici | 136 | 117.6 |
| Tabelout | 437 | 117.6 |
| Humaisi | 593 | 117.6 |
| Bed Al Iguel | 595 | 117.6 |
| Gemlik | 608 | 117.5 |
| Kaissy | 604 | 117.5 |
| Escarabajuelo de Posadas | 235 | 117.5 |
| Escarabajuelo de Úbeda | 236 | 117.5 |
| Djlot Tadmori | 617 | 117.5 |
| Carrasquillo | 335 | 117.4 |
| Ornellaia | 73 | 117.4 |
| Castricianella rapparina | 97 | 117.4 |
| Chetoui | 281 | 117.4 |
| Lechin de Granada | 340 | 117.4 |
| Simjaca | 502 | 117.3 |
| Cirujal | 43 | 117.3 |
| Aîmel | 419 | 117.3 |
| Bolvino | 223 | 117.3 |
| Olivastra di Montalcino | 375 | 117.3 |
| Olivo del Mulino | 165 | 117.3 |
| Olivo de Mancha Real | 260 | 117.3 |
| Rossellino | 60 | 117.3 |
| Mortellino | 373 | 117.3 |
| Masabi | 585 | 117.3 |
| Aaleth | 422 | 117.2 |
| Kothreiki | 176 | 117.2 |
| Tarabelsi | 632 | 117.2 |
| Khashabi-631 | 631 | 117.2 |
| Albatro | 30 | 117.1 |
| Salicino | 71 | 117.1 |
| Negrillo Redondo | 257 | 117.1 |
| Toffahi-621 | 621 | 117.1 |
| Chemchali | 293 | 117.1 |
| Mignolo Cerretano | 46 | 117.1 |
| Craputea | 88 | 117.1 |
| Unkown-OZ1-538 | 538 | 117.1 |
| Unkown-VS5-547 | 547 | 117.1 |
| Racimal | 268 | 117.1 |
| Nasitana Frutto Grosso | 128 | 117.0 |
| Khodieri | 627 | 117.0 |
| Carrasqueno de Elvas | 202 | 117.0 |
| Sayali | 287 | 117.0 |
| Beldi | 288 | 117.0 |
| Hemblasi-601 | 601 | 116.9 |
| Nevado Rizado | 355 | 116.9 |
| Berri Meslal-397 | 397 | 116.9 |
| Idleb | 591 | 116.9 |
| Cassanese | 14 | 116.9 |
| Nocellara del Belice | 129 | 116.9 |
| Souidi | 458 | 116.9 |
| Ciciarello | 2 | 116.9 |
| Brandofino | 86 | 116.8 |
| Puntoza | 499 | 116.8 |
| Negrillo de Estepa | 351 | 116.8 |
| Kossiem | 496 | 116.8 |
| Sebhawy | 492 | 116.8 |
| Kerdi | 597 | 116.8 |
| Azeboudj de Khirane | 445 | 116.7 |
| Cuoricino | 35 | 116.7 |
| Galega Vulgar | 205 | 116.7 |
| Mavreya | 179 | 116.7 |
| Kato Drys | 316 | 116.7 |
| Uovo di Piccione | 141 | 116.6 |
| Limoncillo | 341 | 116.6 |
| Zaity | 603 | 116.5 |
| Manzanilla de Agua | 345 | 116.5 |
| Ronde de la Menara | 543 | 116.5 |
| Sukkare | 663 | 116.5 |
| Buga | 398 | 116.5 |
| Mignolo | 49 | 116.5 |
| Chemlal de Kabilye | 420 | 116.5 |
| Aitana | 78 | 116.5 |
| Crnica-399 | 399 | 116.5 |
| Abiad Min Omou | 616 | 116.4 |
| Lazzero | 25 | 116.4 |
| Bosana | 143 | 116.4 |
| Biancolilla-83 | 83 | 116.4 |
| Maiatica di Ferrandina | 4 | 116.4 |
| San Francesco | 64 | 116.4 |
| Cucca | 32 | 116.4 |
| Akenane | 459 | 116.4 |
| Sant Agostino | 146 | 116.3 |
| Kolybada | 174 | 116.3 |
| Storta | 406 | 116.3 |
| Gerboui | 409 | 116.3 |
| Nocellara Etnea | 126 | 116.3 |
| Jlot | 618 | 116.3 |
| Velika Lastovka | 515 | 116.3 |
| Aggezi Oshime | 482 | 116.2 |
| Aggezi Akse | 481 | 116.2 |
| Zeletni | 421 | 116.2 |
| Unkown-VS1-544 | 544 | 116.2 |
| Bez El Anza | 495 | 116.2 |
| Mohazam Abou Satl | 614 | 116.1 |
| Verdial de Huevar | 213 | 116.1 |
| Picholine Marocaine | 540 | 116.1 |
| Cavalieri | 99 | 116.1 |
| Teffah | 427 | 116.1 |
| Cariasina | 91 | 116.1 |
| Khnfse | 651 | 116.1 |
| Grossa di Spagna | 107 | 116.1 |
| Rechino | 269 | 116.0 |
| Negrillo de Iznalloz | 352 | 116.0 |
| Varudo | 273 | 116.0 |
| Azeradj | 431 | 116.0 |
| Pidicuddara | 157 | 116.0 |
| Koutsourelia | 177 | 115.9 |
| Maraki | 485 | 115.9 |
| Beladi | 573 | 115.9 |
| Wateken | 484 | 115.9 |
| Loaime | 344 | 115.9 |
| Beladi-577 | 577 | 115.8 |
| Baid El Hamam | 491 | 115.8 |
| Ojo de Liebre | 259 | 115.8 |
| Nerba | 123 | 115.8 |
| Lumiaro | 113 | 115.8 |
| Negral de Sabinan-255 | 255 | 115.8 |
| Morisca | 254 | 115.8 |
| Royal de Cazorla | 270 | 115.8 |
| Verdiell | 279 | 115.8 |
| Intosso | 9 | 115.8 |
| Habichuelero de Grazalema | 239 | 115.7 |
| Patronet | 263 | 115.7 |
| Moresca | 121 | 115.7 |
| Berri Meslal-532 | 532 | 115.7 |
| Machorron | 247 | 115.7 |
| Ronde de Miliana | 428 | 115.7 |
| Giarraffa | 105 | 115.7 |
| Ifiri | 457 | 115.7 |
| Unkown-OT2-537 | 537 | 115.7 |
| Cima di Melfi | 92 | 115.7 |
| Barouni | 410 | 115.7 |
| Manzanilla de Montefrio | 250 | 115.6 |
| Bottone di gallo | 84 | 115.6 |
| Villalonga | 201 | 115.6 |
| Gordal de Granada | 238 | 115.6 |
| Mastoidis | 178 | 115.6 |
| El Lewa | 494 | 115.5 |
| Grossane-194 | 194 | 115.5 |
| Morona | 246 | 115.5 |
| Tonda Iblea | 12 | 115.5 |
| Alameno Blanco | 216 | 115.4 |
| Ensasi | 599 | 115.4 |
| Nevado Azul | 354 | 115.4 |
| Trillo | 382 | 115.4 |
| Manzanilla de Hellin | 346 | 115.4 |
| Cordovil de Serpa | 204 | 115.4 |
| Palomar | 262 | 115.4 |
| Ravece | 140 | 115.4 |
| Aglandau | 187 | 115.3 |
| Chalkidikis | 168 | 115.3 |
| Rougette de Mitidja | 444 | 115.3 |
| Mollar de Cieza | 348 | 115.3 |
| Cerasuola | 94 | 115.3 |
| Zalmati-299 | 299 | 115.3 |
| Confetto | 89 | 115.3 |
| Llumeta | 343 | 115.3 |
| Enagua de Arenas | 336 | 115.2 |
| Carolea | 5 | 115.2 |
| Callosina | 391 | 115.2 |
| Vaddarica | 164 | 115.1 |
| Chorruo | 229 | 115.1 |
| Changlot Real | 227 | 115.1 |
| Rapasayo | 357 | 115.1 |
| Jaropo | 242 | 115.1 |
| Gordal Sevillana | 108 | 115.1 |
| Rossello | 62 | 115.0 |
| Empeltre | 234 | 115.0 |
| Verdial transmontana | 214 | 115.0 |
| Nevado Basto | 225 | 115.0 |
| Carrasqueno de Jumilla | 226 | 115.0 |
| Cobrancosa | 203 | 114.9 |
| Akerma | 418 | 114.9 |
| Abunara | 79 | 114.9 |
| Mancanilha Algarvia | 207 | 114.9 |
| Besbessi | 464 | 114.9 |
| Acebuchera | 215 | 114.8 |
| Cornicabra | 232 | 114.8 |
| Verdala | 278 | 114.8 |
| Aggezi Shami | 480 | 114.7 |
| Coratina | 101 | 114.7 |
| El Salam | 488 | 114.7 |
| Aguenaou | 423 | 114.7 |
| Pico Limon de Grazalema | 265 | 114.7 |
| Kalokerida | 171 | 114.7 |
| Unkown-VS2-545 | 545 | 114.7 |
| Crastu | 96 | 114.6 |
| Amargoso | 219 | 114.6 |
| Hojiblanca | 240 | 114.6 |
| Sevillenca | 358 | 114.6 |
| Canivano Negro | 224 | 114.5 |
| Corbella | 230 | 114.5 |
| Bouchouk Lafayette | 430 | 114.5 |
| Negrita | 210 | 114.4 |
| Zarza | 280 | 114.4 |
| Bouteillan | 189 | 114.4 |
| Picholine | 196 | 114.4 |
| Neb Jmel-283 | 283 | 114.4 |
| Ocal | 258 | 114.4 |
| Sevillano de Jumilla | 272 | 114.3 |
| Jabaluna | 241 | 114.3 |
| Bouchouika | 394 | 114.3 |
| Vasilikada | 186 | 114.3 |
| Manzanilla de Sevilla | 251 | 114.3 |
| Morrut | 350 | 114.3 |
| Tempranillo de Yeste-274 | 274 | 114.3 |
| Gerboui-298 | 298 | 114.3 |
| Cayon | 191 | 114.3 |
| Cornezuelo de Jaen | 231 | 114.3 |
| Lechin de Sevilla | 243 | 114.2 |
| Meslala | 535 | 114.1 |
| Alameno de Montilla | 218 | 114.1 |
| Bical | 333 | 114.1 |
| Azul | 221 | 114.0 |
| Lucques | 195 | 114.0 |
| Cairo 7 | 493 | 114.0 |
| Vera | 276 | 113.9 |
| Karolia | 172 | 113.9 |
| Verdial de Badajoz | 342 | 113.9 |
| Dulzal | 233 | 113.9 |
| Karydolia | 173 | 113.8 |
| Lastovka | 506 | 113.7 |
| Varudo-275 | 275 | 113.6 |
| Verdale | 199 | 113.6 |
| Baladi | 487 | 113.6 |
| Redondal | 211 | 113.5 |
| Arbequina | 220 | 113.4 |
| Salonenque | 197 | 113.3 |
| Manzanilla Cacerena | 248 | 113.3 |
| Abou Monkar | 497 | 113.1 |
| Lloron de Atarfe | 245 | 113.1 |
| Toffahi-486 | 486 | 112.7 |
| Agrarez | 436 | 112.7 |
| Dressi | 286 | 112.6 |
| Farga | 338 | 112.6 |
| Fulla de Salze | 339 | 112.2 |
| Sabatera | 271 | 112.2 |
| Madural-208 | 208 | 111.7 |
| Borriolenca | 334 | 110.8 |
