# Supplementary material for: Genome-wide association analysis of flowering date in a collection of cultivated olive tree
Source: Hortic Res. 2024 Sep 24;12(1):uhae265. doi: 10.1093/hr/uhae265 (PMC11718396; doi:10.1093/hr/uhae265)
Supplement: Web_Material_uhae265 [file web_material_uhae265.zip › Aqbouch_etal_Table_S8.docx]

|  |  |  |  |  | MM4LMM Model |  | MLMM Model |  | Sig_SNP |
| --- | --- | --- | --- | --- | --- | --- | --- | --- | --- |
| SNP_name | Linkage group | Position | Alleles(Ref/ALT) | MAF | Weir & Goudet Kinship | VanRaden Kinship | Weir & Goudet Kinship | VanRaden Kinship |  |
| Oe9_LG01_9017729 | Chromosome 01 | 9017729 |  |  | 2.22E-01 |  |  |  |  |
| *Oe9_LG01_9017771* | *Chromosome 01* | *9017771* | *T/C* | *0.17* | *1.50E-07* |  | *1.78E-06* |  | *X* |
| Oe9_LG04_16512341 | Chromosome 04 | 16512341 | G/A | 0.06 | 1.67E-06 |  |  |  | X |
| Oe9_LG04_16512411 | Chromosome 04 | 16512411 | G/C | 0.06 | 1.01E-06 |  | 3.74E-08 | 6.41E-06 | X |
| *Oe9_LG05_12679503* | *Chromosome 05* | *12679503* | *T/C* | *0.22* | *9.60E-06* |  |  |  | *X* |
| Oe9_LG15_5056542 | Chromosome 15 | 5056542 | G/T | 0.14 | 4.33E-06 |  |  |  | X |
| Oe9_LG15_5074116 | Chromosome 15 | 5074116 | A/T | 0.2 | 6.16E-06 |  |  |  | X |
| Oe9_LG15_5074141 | Chromosome 15 | 5074141 | A/G | 0.2 | 4.55E-06 |  |  |  | X |
| Oe9_LG15_5074151 | Chromosome 15 | 5074151 | A/C | 0.2 | 4.55E-06 |  |  |  | X |
| Oe9_LG15_5074198 | Chromosome 15 | 5074198 | G/T | 0.2 | 2.37E-06 |  |  |  | X |
| Oe9_LG19_688809 | Chromosome 19 | 688809 | G/A | 0.18 | 4.22E-06 |  |  |  | X |
| Oe9_LG19_688907 | Chromosome 19 | 688907 | G/A | 0.18 | 6.13E-06 |  |  |  | X |
| Oe9_s00457_64752 | s00457 | 64752 | A/T | 0.18 | 8.88E-06 |  |  |  | X |
| Oe9_s00457_64776 | s00457 | 64776 | C/T | 0.18 | 8.88E-06 |  |  |  | X |
| Oe9_s00457_64781 | s00457 | 64781 | G/A | 0.18 | 4.96E-06 |  |  |  | X |
| Oe9_s00457_64799 | s00457 | 64799 | C/T | 0.18 | 4.96E-06 |  |  |  | X |
| Oe9_s00457_64871 | s00457 | 64871 | C/A | 0.18 | 2.85E-06 |  |  |  | X |
| Oe9_s02016_146839 | s02016 | 146839 |  |  | 2.10E-01 |  |  |  |  |
| Oe9_s02016_146843 | s02016 | 146843 |  |  | 1.36E-04 |  |  |  |  |
| *Oe9_s02016_146890* | *s02016* | *146890* | *G/C* | *0.19* | *7.45E-06* |  |  |  | *X* |
| Oe9_s02016_146952 | s02016 | 146952 |  |  | 1.83E-05 |  |  |  |  |
| Oe9_s02016_147034 | s02016 | 147034 |  |  | 1.83E-05 |  |  |  |  |
| Oe9_s02016_147044 | s02016 | 147044 |  |  | 2.10E-01 |  |  |  |  |
| Oe9_s02016_147069 | s02016 | 147069 |  |  | 5.22E-04 |  |  |  |  |
| Oe9_s02108_269429 | s02108 | 269429 | A/C | 0.33 | 7.97E-06 |  |  |  | X |
| Oe9_s02108_351747 | s02108 | 351747 | A/T | 0.15 | 6.13E-07 |  |  |  | X |
| Oe9_s03956_389829 | s03956 | 389829 | A/G | 0.06 |  |  | 2.72E-06 |  | X |
| Oe9_s04305_16232 | s04305 | 16232 | C/T | 0.1 | 5.10E-06 |  |  |  | X |
| Oe9_s04305_16459 | s04305 | 16459 | T/C | 0.1 | 5.77E-07 |  |  | 1.51E-06 | X |
| Oe9_s04305_16520 | s04305 | 16520 | C/A | 0.1 | 3.00E-06 |  |  |  | X |
| Oe9_s05787_96839 | s05787 | 96839 |  |  | 1.08E-01 |  |  |  |  |
| Oe9_s05787_96859 | s05787 | 96859 |  |  | 6.70E-01 |  |  |  |  |
| Oe9_s05787_96970 | s05787 | 96970 |  |  | 6.25E-01 |  |  |  |  |
| *Oe9_s05787_96972* | *s05787* | *96972* | *G/A* | *0.06* | *2.35E-06* |  |  |  | *X* |
| Oe9_s05787_96986 | s05787 | 96986 |  |  | 6.05E-01 |  |  |  |  |
| Oe9_s06150_161951 | s06150 | 161951 | A/G | 0.07 |  |  | 9.11E-06 |  | X |
| Oe9_s07747_163567 | s07747 | 163567 | T/C | 0.14 |  |  |  | 4.81E-08 | X |
