# Supplementary material for: Genome-wide association analysis of flowering date in a collection of cultivated olive tree
Source: Hortic Res. 2024 Sep 24;12(1):uhae265. doi: 10.1093/hr/uhae265 (PMC11718396; doi:10.1093/hr/uhae265)
Supplement: Web_Material_uhae265 [file web_material_uhae265.zip › Aqbouch_etal_Table_S9.docx]

| Linkage group | Overlap_start | Overlap_end | Significant_SNP_position | Gene_start | Gene_end | Transcrit_name | Gene_ID | Annotation | Ontology_term |
| --- | --- | --- | --- | --- | --- | --- | --- | --- | --- |
| Chromosome 01 | 9017717 | 9019271 | 9017771 | 9017718 | 9022199 | OE9A057547T1 | OE9A057547 | InterPro:IPR007653,Pfam:PF04573 | GO:0005787,GO:0006465,GO:0008233,GO:0016021,GO:0045047 |
| Chromosome 01 | 9017717 | 9019271 | 9017771 | 9017718 | 9022199 | OE9A057547T2 | OE9A057547 | InterPro:IPR007653,Pfam:PF04573 | GO:0005787,GO:0006465,GO:0008233,GO:0016021,GO:0045047 |
| Chromosome 01 | 9017717 | 9019271 | 9017771 | 9017718 | 9022199 | OE9A057547T3 | OE9A057547 | InterPro:IPR007653,PIRSF:PIRSF016089 | GO:0005787,GO:0006465,GO:0008233,GO:0016021,GO:0045047 |
| Chromosome 05 | 12680934 | 12681003 |  | 12680935 | 12688211 | OE9A106916T1 | OE9A106916 | Gene3D:1.10.600.10,Gene3D:1.50.10.130,InterPro:IPR001906,InterPro:IPR005630,InterPro:IPR008930,InterPro:IPR008949,InterPro:IPR036965,InterPro:IPR044814,SUPERFAMILY:SSF48576,Pfam:PF03936,SUPERFAMILY:SSF48239,Pfam:PF01397 | GO:0000287,GO:0009507,GO:0010333,GO:0016102,GO:0016829,GO:0034009 |
| Chromosome 15 | 5056047 | 5058042 | 5056542 | 5056048 | 5058046 | OE9A050487T1 | OE9A050487 | Gene3D:1.10.10.10,Gene3D:3.40.50.150,InterPro:IPR001077,InterPro:IPR012967,InterPro:IPR016461,InterPro:IPR029063,InterPro:IPR036388,InterPro:IPR036390,ProSiteProfiles:PS51683,Pfam:PF08100,SUPERFAMILY:SSF53335,Pfam:PF00891,SUPERFAMILY:SSF46785 | GO:0005737,GO:0008168,GO:0008171,GO:0008757,GO:0017096,GO:0019438,GO:0030187,GO:0032259,GO:0046983 |
| Chromosome 15 | 5056047 | 5058042 | 5056542 | 5056048 | 5058046 | OE9A050487T2 | OE9A050487 | Gene3D:1.10.10.10,Gene3D:3.40.50.150,InterPro:IPR001077,InterPro:IPR012967,InterPro:IPR016461,InterPro:IPR029063,InterPro:IPR036388,InterPro:IPR036390,Pfam:PF00891,PIRSF:PIRSF005739,SUPERFAMILY:SSF46785,SUPERFAMILY:SSF53335,Pfam:PF08100 | GO:0005737,GO:0008168,GO:0008171,GO:0008757,GO:0017096,GO:0019438,GO:0030187,GO:0032259,GO:0046983 |
| Chromosome 15 | 5073813 | 5075698 | 5074116, 5074141, 5074151,5074198 | 5073814 | 5078019 | OE9A037893T6 | OE9A037893 | InterPro:IPR000719,InterPro:IPR002048,InterPro:IPR008271,InterPro:IPR011009,InterPro:IPR011992,InterPro:IPR017441,InterPro:IPR018247,SMART:SM00054,SMART:SM00220,ProSitePatterns:PS00018,ProSitePatterns:PS00107,SUPERFAMILY:SSF47473,SUPERFAMILY:SSF56112,ProSitePatterns:PS00108 | GO:0004672,GO:0004683,GO:0004712,GO:0005509,GO:0005516,GO:0005524,GO:0005634,GO:0005737,GO:0006468,GO:0009931,GO:0016020,GO:0018105,GO:0035556,GO:0046777,GO:0106310 |
| Chromosome 15 | 5073813 | 5075698 | 5074116, 5074141, 5074151,5074198 | 5073814 | 5078019 | OE9A037893T5 | OE9A037893 | InterPro:IPR000719,InterPro:IPR002048,InterPro:IPR008271,InterPro:IPR011009,InterPro:IPR011992,InterPro:IPR017441,InterPro:IPR018247,SMART:SM00054,SMART:SM00220,ProSitePatterns:PS00018,ProSitePatterns:PS00107,SUPERFAMILY:SSF47473,SUPERFAMILY:SSF56112,ProSitePatterns:PS00108 | GO:0004672,GO:0004683,GO:0004712,GO:0005509,GO:0005516,GO:0005524,GO:0005634,GO:0005737,GO:0006468,GO:0009931,GO:0016020,GO:0018105,GO:0035556,GO:0046777,GO:0106310 |
| Chromosome 15 | 5073840 | 5075698 | 5074116, 5074141, 5074151,5074198 | 5073841 | 5078862 | OE9A037893T4 | OE9A037893 | InterPro:IPR000719,InterPro:IPR002048,InterPro:IPR008271,InterPro:IPR011009,InterPro:IPR011992,InterPro:IPR017441,InterPro:IPR018247,ProSiteProfiles:PS50222,SUPERFAMILY:SSF47473,SMART:SM00220,ProSitePatterns:PS00018,ProSitePatterns:PS00107,SUPERFAMILY:SSF56112,ProSitePatterns:PS00108 | GO:0004672,GO:0004674,GO:0005509,GO:0005524,GO:0006468 |
| Chromosome 15 | 5073813 | 5075698 | 5074116, 5074141, 5074151,5074198 | 5073814 | 5080078 | OE9A037893T2 | OE9A037893 | InterPro:IPR000719,InterPro:IPR002048,InterPro:IPR008271,InterPro:IPR011009,InterPro:IPR011992,InterPro:IPR017441,InterPro:IPR018247,SMART:SM00054,ProSitePatterns:PS00018,SMART:SM00220,ProSitePatterns:PS00107,SUPERFAMILY:SSF47473,SUPERFAMILY:SSF56112,ProSitePatterns:PS00108 | GO:0004672,GO:0004683,GO:0004712,GO:0005509,GO:0005516,GO:0005524,GO:0005634,GO:0005737,GO:0006468,GO:0009931,GO:0016020,GO:0018105,GO:0035556,GO:0046777,GO:0106310 |
| Chromosome 15 | 5073813 | 5075698 | 5074116, 5074141, 5074151,5074198 | 5073814 | 5080078 | OE9A037893T3 | OE9A037893 | InterPro:IPR000719,InterPro:IPR002048,InterPro:IPR008271,InterPro:IPR011009,InterPro:IPR011992,InterPro:IPR017441,InterPro:IPR018247,ProSiteProfiles:PS50222,SUPERFAMILY:SSF47473,SMART:SM00220,ProSitePatterns:PS00018,ProSitePatterns:PS00107,SUPERFAMILY:SSF56112,ProSitePatterns:PS00108 | GO:0004672,GO:0004674,GO:0005509,GO:0005524,GO:0006468 |
| Chromosome 15 | 5073840 | 5075698 | 5074116, 5074141, 5074151,5074198 | 5073841 | 5080078 | OE9A037893T1 | OE9A037893 | InterPro:IPR000719,InterPro:IPR002048,InterPro:IPR008271,InterPro:IPR011009,InterPro:IPR011992,InterPro:IPR017441,InterPro:IPR018247,ProSiteProfiles:PS50222,ProSitePatterns:PS00018,SUPERFAMILY:SSF47473,SMART:SM00220,ProSitePatterns:PS00107,SUPERFAMILY:SSF56112,ProSitePatterns:PS00108 | GO:0004672,GO:0004674,GO:0005509,GO:0005524,GO:0006468 |
| Chromosome 19 | 688608 | 690407 | 688809, 688907 | 688609 | 691463 | OE9A121123T1 | OE9A121123 | Gene3D:3.30.70.80,Gene3D:3.40.50.200,InterPro:IPR000209,InterPro:IPR003137,InterPro:IPR010259,InterPro:IPR015500,InterPro:IPR023828,InterPro:IPR034197,InterPro:IPR036852,InterPro:IPR037045,InterPro:IPR041469,InterPro:IPR045051,Pfam:PF00082,Pfam:PF17766,ProSitePatterns:PS00138,PRINTS:PR00723,Pfam:PF05922,Pfam:PF02225,CDD:cd04852 | GO:0004252,GO:0005886,GO:0006508,GO:0008236,GO:0009827 |
| s00457 | 64506 | 66371 | 64752, 64776, 64781, 64799, 64871 | 64507 | 70331 | OE9A111219T1 | OE9A111219 | InterPro:IPR001509,InterPro:IPR010099,InterPro:IPR013549,InterPro:IPR036291,TIGRFAM:TIGR01777,Pfam:PF08338,SUPERFAMILY:SSF51735,Pfam:PF01370 | GO:0009507,GO:0009536,GO:0009706,GO:0009941,GO:0010020,GO:0016491,GO:0042803 |
| s02016 | 146687 | 148390 | 146890 | 146688 | 149129 | OE9A056765T1 | OE9A056765 | InterPro:IPR001810,InterPro:IPR025886,InterPro:IPR036047,Pfam:PF14299,SUPERFAMILY:SSF81383 | GO:0005515,GO:0009793 |
| s02108 | 268097 | 268436 |  | 268098 | 268436 | OE9A042265T1 | OE9A042265 |  |  |
| s02108 | 269113 | 270929 | 269429 | 269114 | 275657 | OE9A068749T1 | OE9A068749 | Gene3D:1.20.1250.20,InterPro:IPR001958,InterPro:IPR011701,InterPro:IPR020846,InterPro:IPR036259,Pfam:PF07690,SUPERFAMILY:SSF103473 | GO:0006817,GO:0016021,GO:0022857,GO:0050896,GO:0055085 |
| s02108 | 267929 | 269385 |  | 262020 | 269385 | OE9A000738T1 | OE9A000738 | Gene3D:1.25.40.10,InterPro:IPR002885,InterPro:IPR011990,InterPro:IPR045215,Pfam:PF13041 | GO:0003723,GO:0005515,GO:0005739,GO:0009451,GO:0016554,GO:0043231 |
| s02108 | 351537 | 353247 | 351747 | 351538 | 355380 | OE9A042687T1 | OE9A042687 | Gene3D:3.30.710.10,InterPro:IPR001232,InterPro:IPR011333,InterPro:IPR016072,InterPro:IPR016073,InterPro:IPR016897,InterPro:IPR036296,SUPERFAMILY:SSF81382,PIRSF:PIRSF028729,SMART:SM00512,Pfam:PF03931,SUPERFAMILY:SSF54695,Pfam:PF01466 | GO:0000226,GO:0005634,GO:0005737,GO:0005739,GO:0005819,GO:0005829,GO:0006511,GO:0007059,GO:0009524,GO:0009733,GO:0009734,GO:0009753,GO:0009867,GO:0009873,GO:0016567,GO:0019005,GO:0031146,GO:0045910,GO:0097602 |
| s04305 | 14732 | 16127 |  | 14465 | 16127 | OE9A117378T1 | OE9A117378 | InterPro:IPR007005,PANTHER:PTHR12722 | GO:0005634,GO:0048511 |
| s04305 | 16130 | 18020 | 16232, 16459, 16520 | 16131 | 18668 | OE9A084268T1 | OE9A084268 | InterPro:IPR007005,PANTHER:PTHR12722 | GO:0005634,GO:0006325,GO:0009637,GO:0009873,GO:0010099,GO:0010114,GO:0035196,GO:0042752,GO:0048511 |
| s05787 | 96603 | 98472 | 96972 | 96604 | 99620 | OE9A051761T1 | OE9A051761 | Gene3D:1.20.140.40,Gene3D:2.160.20.10,InterPro:IPR000070,InterPro:IPR006501,InterPro:IPR011050,InterPro:IPR012334,InterPro:IPR033131,InterPro:IPR035513,Pfam:PF04043,Pfam:PF01095,ProSitePatterns:PS00503,SUPERFAMILY:SSF51126 | GO:0004857,GO:0016021,GO:0030599,GO:0042545,GO:0045330,GO:0045490 |
| s05787 | 96634 | 98472 | 96972 | 96635 | 99463 | OE9A051761T2 | OE9A051761 | Gene3D:1.20.140.40,Gene3D:2.160.20.10,InterPro:IPR000070,InterPro:IPR006501,InterPro:IPR011050,InterPro:IPR012334,InterPro:IPR033131,InterPro:IPR035513,Pfam:PF04043,SUPERFAMILY:SSF51126,Pfam:PF01095,ProSitePatterns:PS00503 | GO:0004857,GO:0016021,GO:0030599,GO:0042545,GO:0045330,GO:0045490 |
| s06150 | 161734 | 163451 | 161951 | 161735 | 165718 | OE9A054828T1 | OE9A054828 | InterPro:IPR000047,InterPro:IPR001356,InterPro:IPR003106,InterPro:IPR009057,InterPro:IPR017970,InterPro:IPR045224,ProSitePatterns:PS00027,SMART:SM00389,SUPERFAMILY:SSF46689,PRINTS:PR00031,Pfam:PF02183 | GO:0000981,GO:0003677,GO:0005634,GO:0006355,GO:0043565 |
| s06150 | 161739 | 163451 | 161951 | 161740 | 164471 | OE9A054828T2 | OE9A054828 | InterPro:IPR000047,InterPro:IPR001356,InterPro:IPR003106,InterPro:IPR009057,InterPro:IPR017970,InterPro:IPR045224,ProSitePatterns:PS00027,ProSiteProfiles:PS50071,SUPERFAMILY:SSF46689,PRINTS:PR00031,Pfam:PF02183 | GO:0000981,GO:0003677,GO:0005634,GO:0006355,GO:0043565 |
| s06150 | 161744 | 163451 | 161951 | 161745 | 165676 | OE9A054828T4 | OE9A054828 | InterPro:IPR000047,InterPro:IPR001356,InterPro:IPR003106,InterPro:IPR009057,InterPro:IPR017970,InterPro:IPR045224,SMART:SM00389,SUPERFAMILY:SSF46689,PRINTS:PR00031,Pfam:PF02183,ProSitePatterns:PS00027 | GO:0000981,GO:0003677,GO:0005634,GO:0006355,GO:0043565 |
| s06150 | 161744 | 163451 | 161951 | 161745 | 165676 | OE9A054828T5 | OE9A054828 | InterPro:IPR000047,InterPro:IPR001356,InterPro:IPR003106,InterPro:IPR009057,InterPro:IPR017970,InterPro:IPR045224,SUPERFAMILY:SSF46689,PRINTS:PR00031,CDD:cd00086,Pfam:PF02183,ProSitePatterns:PS00027 | GO:0000981,GO:0003677,GO:0005634,GO:0006355,GO:0043565 |
| s06150 | 161744 | 163451 | 161951 | 161745 | 165676 | OE9A054828T3 | OE9A054828 | InterPro:IPR000047,InterPro:IPR001356,InterPro:IPR003106,InterPro:IPR009057,InterPro:IPR017970,InterPro:IPR045224,SUPERFAMILY:SSF46689,PRINTS:PR00031,CDD:cd00086,Pfam:PF02183,ProSitePatterns:PS00027 | GO:0000981,GO:0003677,GO:0005634,GO:0006355,GO:0043565 |
| s07747 | 162067 | 163181 | 163567 | 161903 | 163181 | OE9A034024T1 | OE9A034024 | Gene3D:2.60.40.790,InterPro:IPR002068,InterPro:IPR008978,InterPro:IPR044656,Pfam:PF00011 | GO:0005739 |
| s07747 | 163590 | 164129 | 163567 | 163591 | 164129 | OE9A073295T1 | OE9A073295 |  |  |
| s03956 | 388659 | 390581 | 389829 | 388660 | 390581 | OE9A113498T1 | OE9A113498 | Gene3D:3.30.40.10,InterPro:IPR001841,InterPro:IPR013083,InterPro:IPR044675,SMART:SM00184 | GO:0016021,GO:0016567 |
