# Supplementary material for: Genome-wide association analysis of flowering date in a collection of cultivated olive tree
Source: Hortic Res. 2024 Sep 24;12(1):uhae265. doi: 10.1093/hr/uhae265 (PMC11718396; doi:10.1093/hr/uhae265)
Supplement: Web_Material_uhae265 [file web_material_uhae265.zip › Aqbouch_etal_Table_S10.docx]

| Transcrit_name | Status | Protein Name | Gene Name | Organism | Percent identity(%) | E value | Function |
| --- | --- | --- | --- | --- | --- | --- | --- |
| OE9A057547T1 | reviewed | Signal peptidase complex subunit 3B | At5g27430 | Arabidopsis thaliana (Mouse-ear cress) | 80.2 | 1.20E-70 | Essential component of the signal peptidase complex (SPC) which catalyzes the cleavage of N-terminal signal sequences from nascent proteins as they are translocated into the lumen of the endoplasmic reticulum (By similarity). Essential for the SPC catalytic activity, possibly by stabilizing and positioning the active center of the complex close to the lumenal surface (By similarity). |
| OE9A057547T2 | reviewed | Signal peptidase complex subunit 3B | At5g27430 | Arabidopsis thaliana (Mouse-ear cress) | 80.2 | 1.70E-70 |  |
| OE9A057547T3 | reviewed | Signal peptidase complex subunit 3B | At5g27430 | Arabidopsis thaliana (Mouse-ear cress) | 80.2 | 6.30E-99 |  |
| OE9A106916T1 | reviewed | Isoprene synthase, chloroplastic | ISPS | Populus tremuloides (Quaking aspen) | 51.6 | 7.10E-131 | Lyase that catalyzes the formation of isoprene from dimethylallyl diphosphate. |
| OE9A050487T1 | unreviewed | (RS)-norcoclaurine 6-O-methyltransferase-like | OLEA9_A050487 | Olea europaea subsp. europaea | 100 | 0 | NA |
| OE9A050487T2 | unreviewed | (RS)-norcoclaurine 6-O-methyltransferase-like | OLEA9_A050487 | Olea europaea subsp. europaea | 100 | 0 | NA |
| OE9A037893T6 | reviewed | Calcium-dependent protein kinase 4 | CPK4 | Solanum tuberosum (Potato) | 83.5 | 0 | Regulates the production of reactive oxygen species (ROS) by NADPH oxidase. |
| OE9A037893T5 | reviewed | Calcium-dependent protein kinase 4 | CPK4 | Solanum tuberosum (Potato) | 83.5 | 0 |  |
| OE9A037893T4 | reviewed | Calcium-dependent protein kinase 4 | CPK4 | Solanum tuberosum (Potato) | 85.2 | 0 |  |
| OE9A037893T2 | reviewed | Calcium-dependent protein kinase 4 | CPK5 | Solanum tuberosum (Potato) | 85.2 | 0 |  |
| OE9A037893T3 | reviewed | Calcium-dependent protein kinase 4 | CPK4 | Solanum tuberosum (Potato) | 85.2 | 0 |  |
| OE9A037893T1 | reviewed | Calcium-dependent protein kinase 4 | CPK4 | Solanum tuberosum (Potato) | 85.2 | 0 |  |
| OE9A121123T1 | reviewed | Subtilisin-like protease SBT1.6 | SBT1.6 | Arabidopsis thaliana (Mouse-ear cress) | 74.4 | 0 | NA |
| OE9A111219T1 | reviewed | Epimerase family protein SDR39U1 homolog, chloroplastic | GC1 | Arabidopsis thaliana (Mouse-ear cress) | 81.3 | 0 | Putative NADP-dependent oxidoreductase that acts as positive regulator of chloroplast division. May play a role at an early stage of the division process. |
| OE9A056765T1 | unreviewed | F-box PP2-B10-like | OLEA9_A056765 | Olea europaea subsp. europaea | 100 | 0 | NA |
| OE9A042265T1 | unreviewed | Uncharacterized protein | OLEA9_A042265 | Olea europaea subsp. europaea | 100 | 0 | NA |
| OE9A068749T1 | unreviewed | Hippocampus abundant transcript 1 | OLEA9_A068749 | Olea europaea subsp. europaea | 100 | 0 | NA |
| OE9A000738T1 | reviewed | Pentatricopeptide repeat-containing protein At1g62260, mitochondrial | PCMP-E10 | Arabidopsis thaliana (Mouse-ear cress) | 59.5 | 0 | NA |
| OE9A042687T1 | unreviewed | SKP1-like protein | OLEA9_A042687 | Olea europaea subsp. europaea | 100 | 0 | Involved in ubiquitination and subsequent proteasomal degradation of target proteins. Together with CUL1, RBX1 and a F-box protein, it forms a SCF E3 ubiquitin ligase complex. The functional specificity of this complex depends on the type of F-box protein. In the SCF complex, it serves as an adapter that links the F-box protein to CUL1. |
| OE9A117378T1 | reviewed | Protein XAP5 CIRCADIAN TIMEKEEPER | XCT | Oryza sativa subsp. japonica (Rice) | 80.1 | 1.10E-79 | Involved in light regulation of the circadian clock and photomorphogenesis. |
| OE9A084268T1 | reviewed | Protein XAP5 CIRCADIAN TIMEKEEPER | XCT | Arabidopsis thaliana (Mouse-ear cress) | 94.8 | 4.00E-118 | Involved in light regulation of the circadian clock and photomorphogenesis. May play a global role in coordinating growth in response to the light environment. Acts as a light quality sensor directing both negative and positive transcriptional regulation. Inhibits growth in red light but promote growth in blue light. Inhibits clock gene expression in diurnal cycles. Plays no role in the control of flowering time |
| OE9A051761T1 | reviewed | Probable pectinesterase/pectinesterase inhibitor 34 | PME34 | Arabidopsis thaliana (Mouse-ear cress) | 64.7 | 0 | Acts in the modification of cell walls via demethylesterification of cell wall pectin. |
| OE9A051761T2 | reviewed | Probable pectinesterase/pectinesterase inhibitor 34 | PME34 | Arabidopsis thaliana (Mouse-ear cress) | 64.4 | 0 |  |
| OE9A054828T1 | unreviewed | Homeobox-leucine zipper protein | OLEA9_A054828 | Olea europaea subsp. europaea | 100 | 9.90E-22 | NA |
| OE9A054828T2 | unreviewed | Homeobox-leucine zipper protein | OLEA9_A054828 | Olea europaea subsp. europaea | 98.6 | 6.40E-86 | NA |
| OE9A054828T4 | unreviewed | Homeobox-leucine zipper protein | OLEA9_A054828 | Olea europaea subsp. europaea | 98.6 | 6.10E-86 | NA |
| OE9A054828T5 | unreviewed | Homeobox-leucine zipper protein | OLEA9_A054828 | Olea europaea subsp. europaea | 98.6 | 6.10E-86 | NA |
| OE9A054828T3 | unreviewed | Homeobox-leucine zipper protein | OLEA9_A054828 | Olea europaea subsp. europaea | 98.6 | 6.10E-86 | NA |
| OE9A034024T1 | reviewed | Heat shock 22 kDa protein, mitochondrial | HSP22 | Pisum sativum (Garden pea) (Lathyrus oleraceus) | 67.9 | 7.20E-50 | NA |
| OE9A073295T1 | reviewed | Uncharacterized histidine-rich protein DDB_G0274557 | DDB_G0274557 | Dictyostelium discoideum (Social amoeba) | 27.2 | 0.053 | NA |
| OE9A113498T1 | unreviewed | RING-type E3 ubiquitin transferase | OLEA9_A113498 | Olea europaea subsp. europaea | 100 | 0 | NA |
