# Supplementary material for: Genome-wide association analysis of flowering date in a collection of cultivated olive tree
Source: Hortic Res. 2024 Sep 24;12(1):uhae265. doi: 10.1093/hr/uhae265 (PMC11718396; doi:10.1093/hr/uhae265)
Supplement: Web_Material_uhae265 [file web_material_uhae265.zip › Aqbouch_etal_Table_S11.docx]

| Genetic_group | ssr_C1 | ssr_C2 | ssr_C3 | ssr_M | **Total général** |
| --- | --- | --- | --- | --- | --- |
| C1 | 43 | 2 |  | 27 | **72** |
| C2 |  | 18 |  | 13 | **31** |
| C3 |  |  | 64 | 5 | **69** |
| M | 1 | 39 | 7 | 78 | **125** |
| **Total général** | **44** | **59** | **71** | **123** | **297** |
|  |  |  |  |  |  |
|  |  |  |  |  |  |
| Genetic_group | East | Center | West | ssr_admixed | **Total général** |
| C1 | 60% | 3% |  | 38% | **72** |
| C2 |  | 58% |  | 42% | **31** |
| C3 |  |  | 93% | 7% | **69** |
| M | 1% | 31% | 6% | 62% | **125** |
| **General concordance** | **68%** | | | |  |
