# Supplementary material for: Genome-wide association analysis of flowering date in a collection of cultivated olive tree
Source: Hortic Res. 2024 Sep 24;12(1):uhae265. doi: 10.1093/hr/uhae265 (PMC11718396; doi:10.1093/hr/uhae265)
Supplement: Web_Material_uhae265 [file web_material_uhae265.zip › Aqbouch_etal_Table_S12.docx]

| Genetic_group | Q3 | Q2 | Q1 | Mosaic | **Total** |
| --- | --- | --- | --- | --- | --- |
| C1 | 9 |  |  | 4 | **13** |
| C2 |  |  |  | 2 | **2** |
| C3 |  |  | 25 | 3 | **28** |
| M |  | 15 | 1 | 14 | **30** |
| **Total général** | **9** | **15** | **26** | **23** | **73** |
|  |  |  |  |  |  |
|  |  |  |  |  |  |
| Genetic_group | Q3 | Q2 | Q1 | Mosaic | **Total** |
| C1 | 69% |  |  | 31% | **13** |
| C2 |  |  |  | 100% | **2** |
| C3 |  |  | 89% | 11% | **28** |
| M |  | 50% | 3% | 47% | **30** |
| **Genral concordance** | **66%** | | | |  |
