# Supplementary material for: Genome-wide association analysis of flowering date in a collection of cultivated olive tree
Source: Hortic Res. 2024 Sep 24;12(1):uhae265. doi: 10.1093/hr/uhae265 (PMC11718396; doi:10.1093/hr/uhae265)
Supplement: Web_Material_uhae265 [file web_material_uhae265.zip › Aqbouch_etal_Table_S13.docx]

| Genetic_group | A | B | C | EST_SNP_M | **Total** |
| --- | --- | --- | --- | --- | --- |
| C1 | 8 |  |  | 3 | **11** |
| C2 |  | 1 |  | 3 | **4** |
| C3 |  |  | 45 | 1 | **46** |
| M |  | 2 | 6 | 30 | **38** |
| **Total général** | **8** | **3** | **51** | **37** | **99** |
|  |  |  |  |  |  |
| Genetic_group | A | B | C | EST_SNP_M | **Total** |
| C1 | 73% |  |  | 27% | **11** |
| C2 |  | 25% |  | 75% | **4** |
| C3 |  |  | 98% | 2% | **46** |
| M |  | 5% | 16% | 79% | **38** |
| **Genral concordance** | **85%** | | | |  |
