# Supplementary material for: Genome-wide association analysis of flowering date in a collection of cultivated olive tree
Source: Hortic Res. 2024 Sep 24;12(1):uhae265. doi: 10.1093/hr/uhae265 (PMC11718396; doi:10.1093/hr/uhae265)
Supplement: Web_Material_uhae265 [file web_material_uhae265.zip › Aqbouch_etal_Table_S14.docx]

| Genetic_group | C'1 | C'2 | C'3 | M' | **Total général** |
| --- | --- | --- | --- | --- | --- |
| C1 | 78 |  |  | 1 | **79** |
| C2 |  | 30 |  | 3 | **33** |
| C3 |  |  | 70 | 1 | **71** |
| M | 4 | 4 | 1 | 126 | **135** |
| **Total général** | **82** | **34** | **71** | **131** | **318** |
|  |  |  |  |  |  |
| Genetic_group | C'1 | C'2 | C'3 | M' | **Total général** |
| C1 | 99% |  |  | 1% | **79** |
| C2 |  | 91% |  | 9% | **33** |
| C3 |  |  | 99% | 1% | **71** |
| M | 3% | 3% | 1% | 93% | **135** |
| **General concordance** | **96%** | | | |  |
