# Supplementary material for: Genome-wide association analysis of flowering date in a collection of cultivated olive tree
Source: Hortic Res. 2024 Sep 24;12(1):uhae265. doi: 10.1093/hr/uhae265 (PMC11718396; doi:10.1093/hr/uhae265)
Supplement: Web_Material_uhae265 [file web_material_uhae265.zip › Aqbouch_etal_Table_S15.docx]

| Genotype | Pourcentage of different loci (based on VCF before filters) | Pourcentage of different loci (based on VCF after filters) |
| --- | --- | --- |
| Leccino | 5.88% | 2.46% |
| Picholine Marocaine | 5.85% | 2.30% |
| Picual | 6.16% | 2.72% |
| Mean | 5.96% | 2.49% |
