# Supplementary material for: Genome-wide association analysis of flowering date in a collection of cultivated olive tree
Source: Hortic Res. 2024 Sep 24;12(1):uhae265. doi: 10.1093/hr/uhae265 (PMC11718396; doi:10.1093/hr/uhae265)
Supplement: Web_Material_uhae265 [file web_material_uhae265.zip › Aqbouch_etal_Table_S16.docx]

| Model | npar | AIC | BIC | logLik | deviance | Chisq | Df | Pr(>Chisq) | Signification |
| --- | --- | --- | --- | --- | --- | --- | --- | --- | --- |
| FFD= \|Genotype | 3 | 53634 | 53655 | -26814 | 53628 |  |  |  |  |
| FFD= \|Genotype + year | 9 | 37099 | 37161 | -18541 | 37081 | 16547.6 | 6 | 2.20E-16 | *** |
| FFD= \|Genotype + year + \|Genotype:year | 10 | 35591 | 35659 | -17785 | 35571 | 1510.4 | 1 | 2.20E-16 | *** |
