# Supplementary material for: Genome-wide association analysis of flowering date in a collection of cultivated olive tree
Source: Hortic Res. 2024 Sep 24;12(1):uhae265. doi: 10.1093/hr/uhae265 (PMC11718396; doi:10.1093/hr/uhae265)
Supplement: Web_Material_uhae265 [file web_material_uhae265.zip › Aqbouch_etal_Table_S17.docx]

|  | Df | Sum of Squares | Mean Square | F value | Pr>F | Signification |
| --- | --- | --- | --- | --- | --- | --- |
| Genotype | 330 | 44110 | 134 | 24.26 | <2e-16 | *** |
| year | 6 | 607453 | 101242 | 18373.95 | <2e-16 | *** |
| Genotype:year | 1436 | 31567 | 22 | 3.99 | <2e-16 | *** |
| Residuals | 5448 | 30019 | 6 |  |  |  |
