# Supplementary figures and images for: Genome-wide association analysis of flowering date in a collection of cultivated olive tree
Source: Hortic Res. 2024 Sep 24;12(1):uhae265. doi: 10.1093/hr/uhae265 (PMC11718396; doi:10.1093/hr/uhae265)

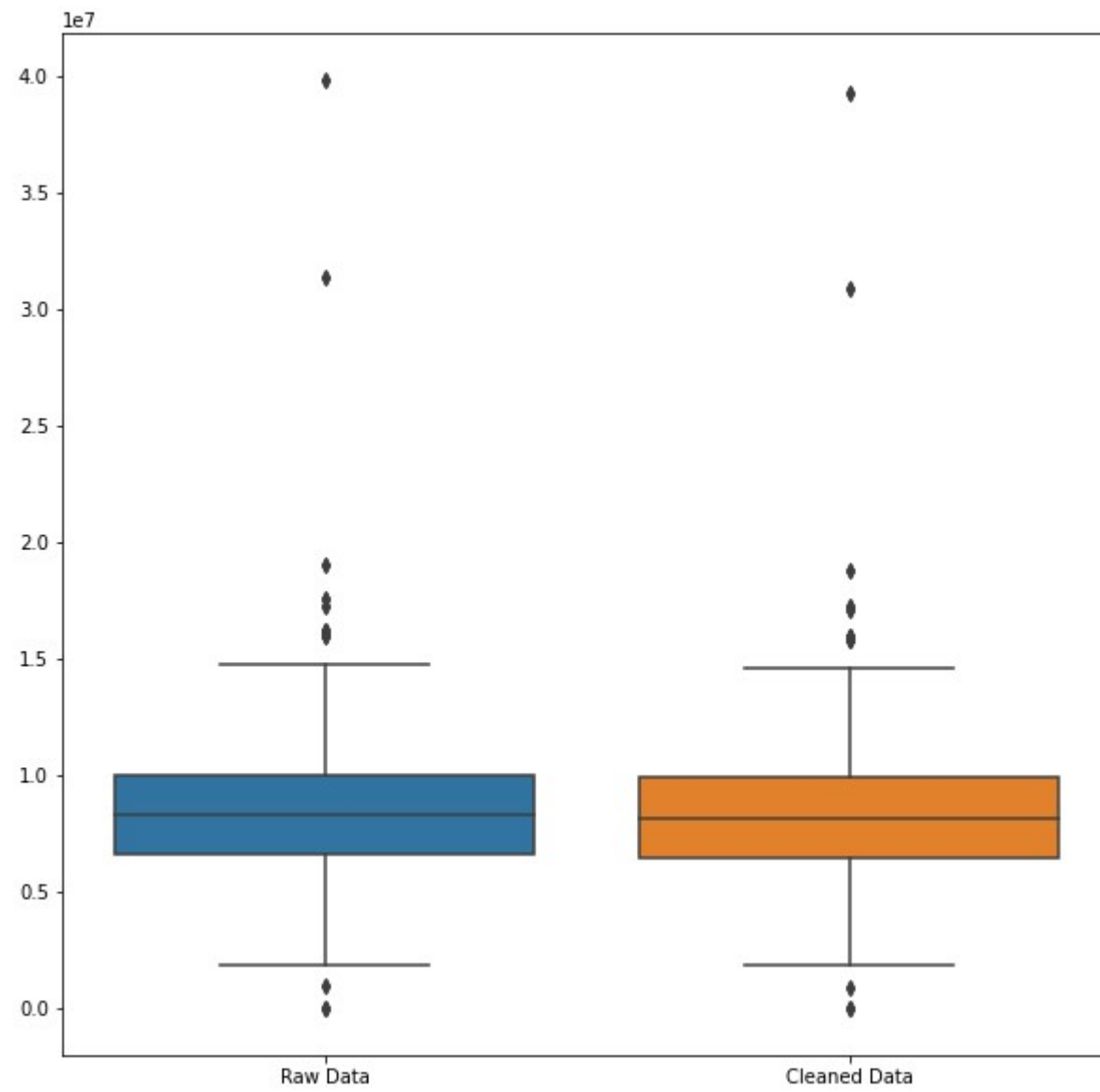

Supplement: Web_Material_uhae265 [file web_material_uhae265.zip › Aqbouch_etal_Figure_S1.pdf]

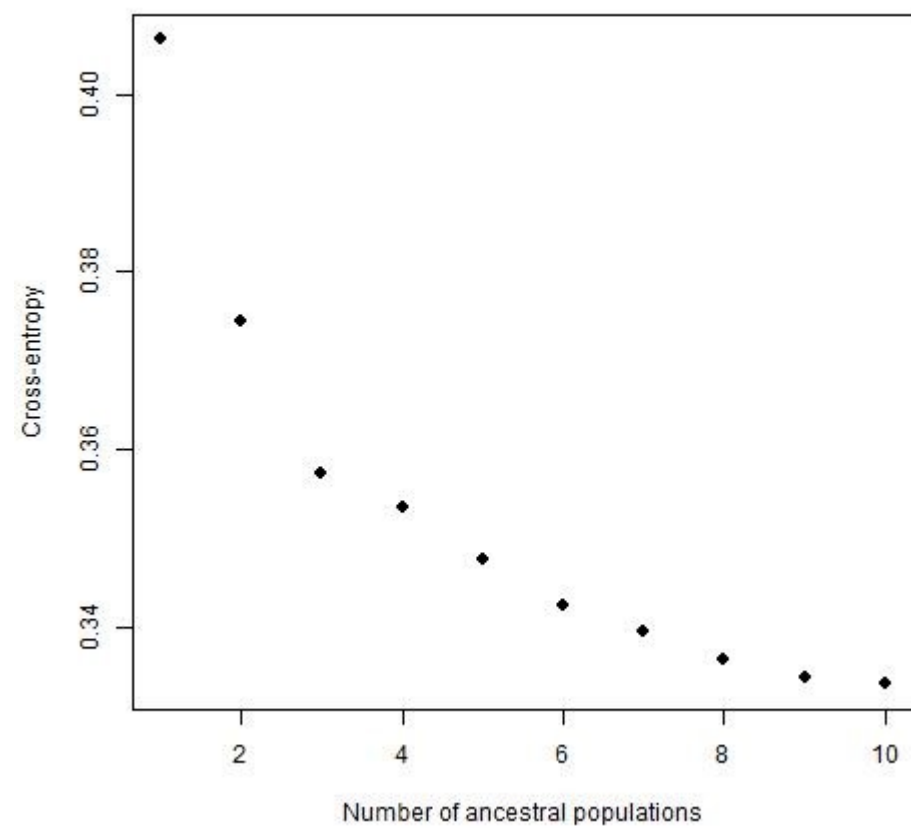

Supplement: Web_Material_uhae265 [file web_material_uhae265.zip › Aqbouch_etal_Figure_S2.pdf]

Scree Plot - K = 10

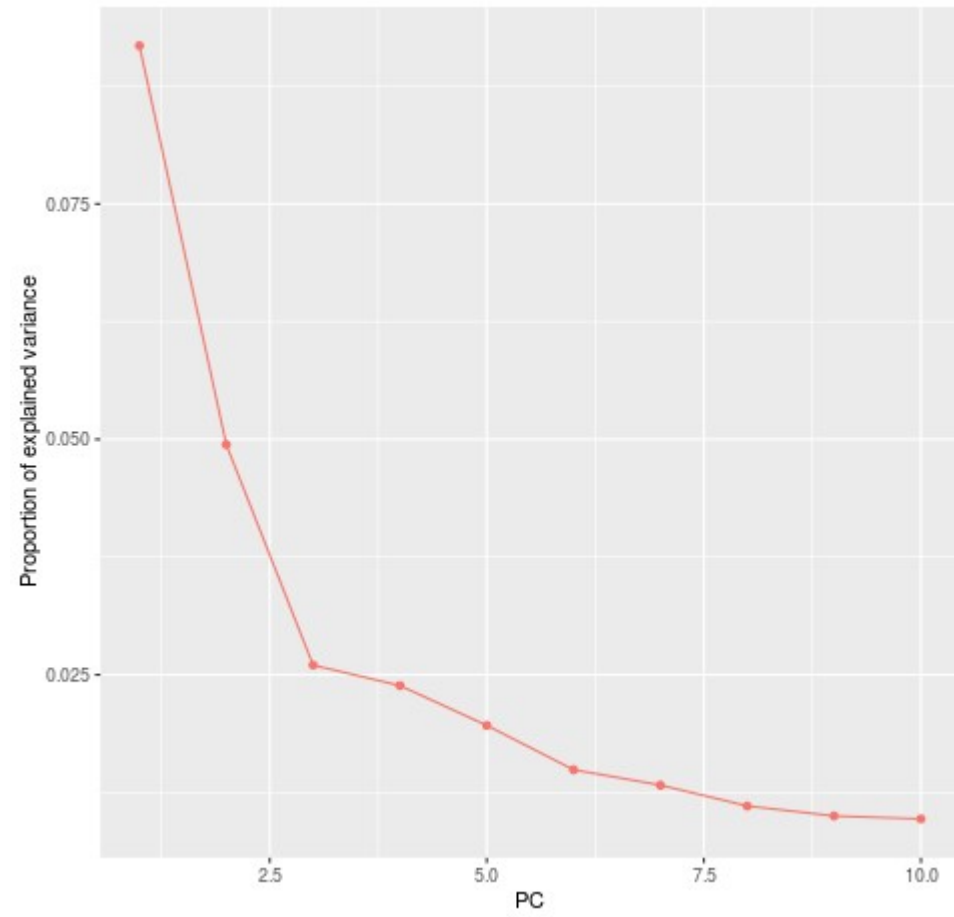

Supplement: Web_Material_uhae265 [file web_material_uhae265.zip › Aqbouch_etal_Figure_S3.pdf]

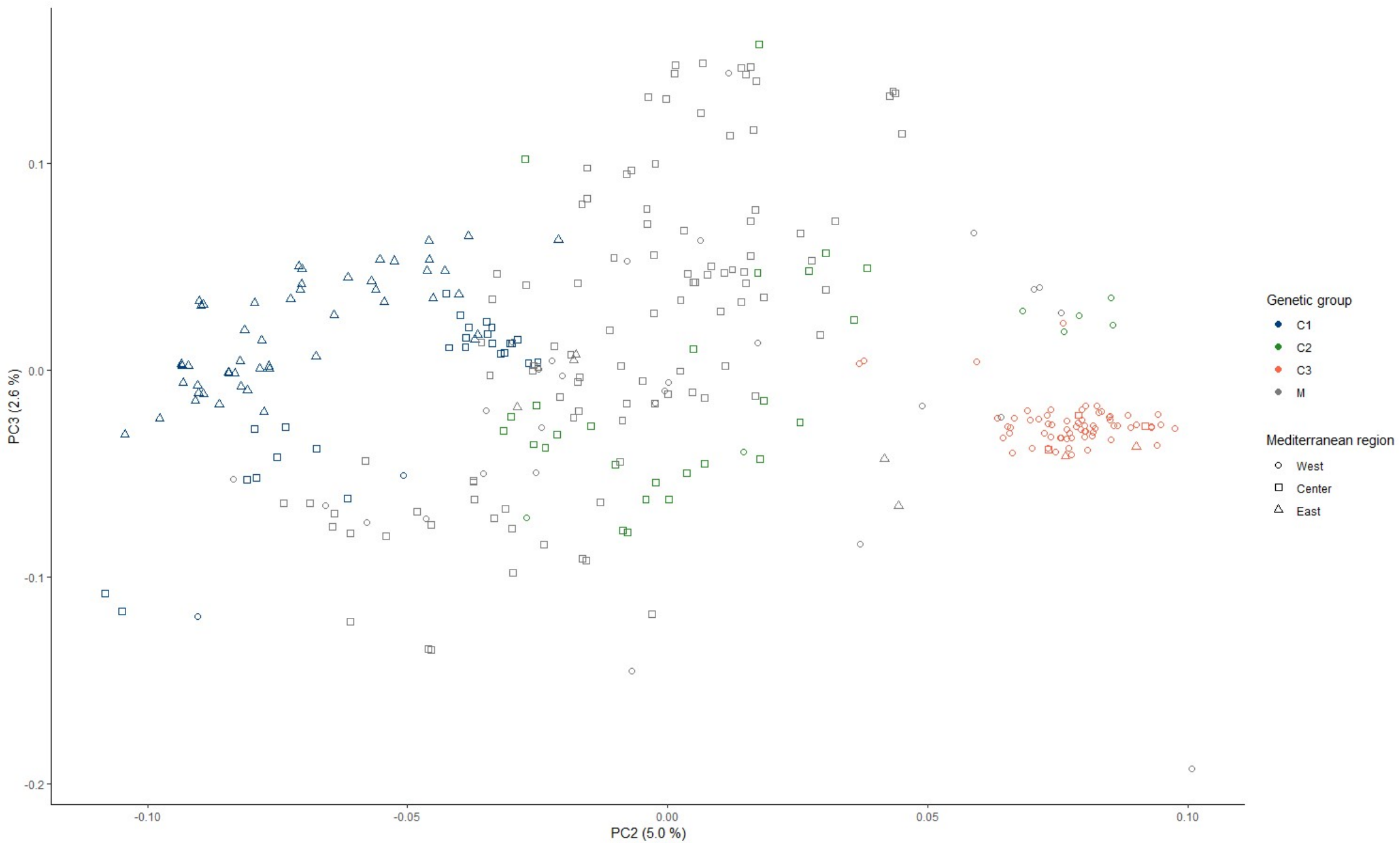

Supplement: Web_Material_uhae265 [file web_material_uhae265.zip › Aqbouch_etal_Figure_S4.pdf]

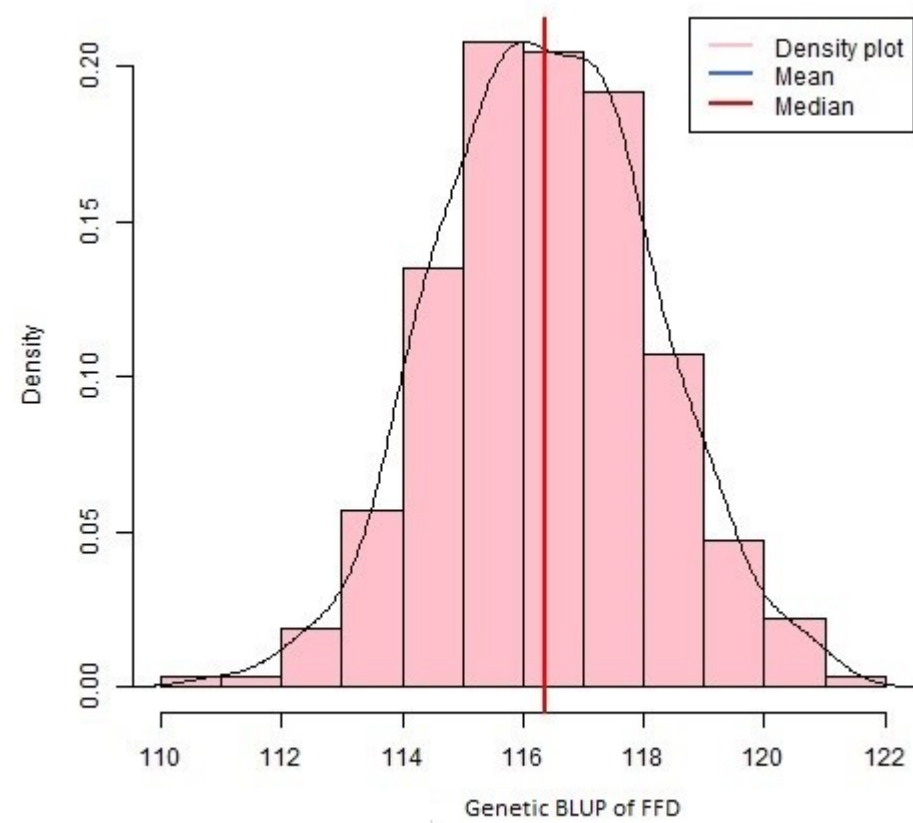

Supplement: Web_Material_uhae265 [file web_material_uhae265.zip › Aqbouch_etal_Figure_S5.pdf]

A

Oe9\_LG01\_9017771

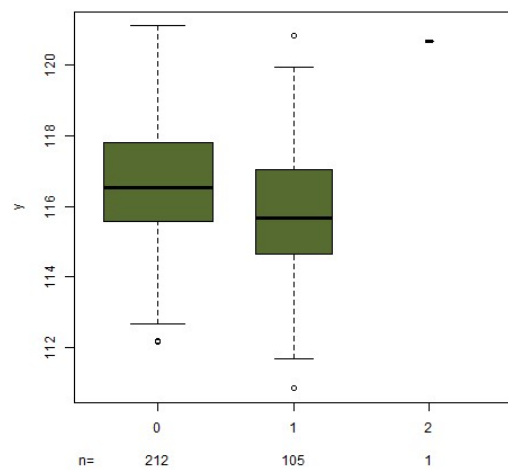

B

Oe9\_LG04\_16512411

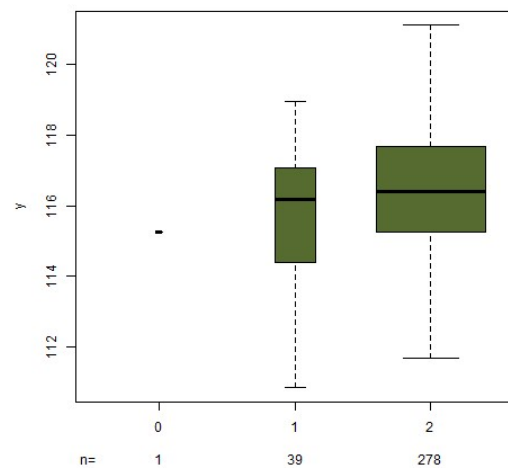

C

Oe9\_s04305\_16459

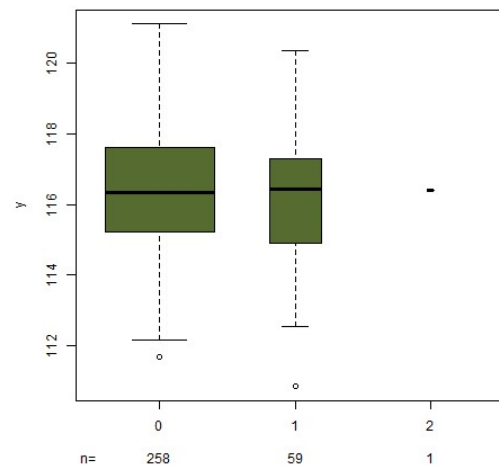

Supplement: Web_Material_uhae265 [file web_material_uhae265.zip › Aqbouch_etal_Figure_S6.pdf]

LD decay

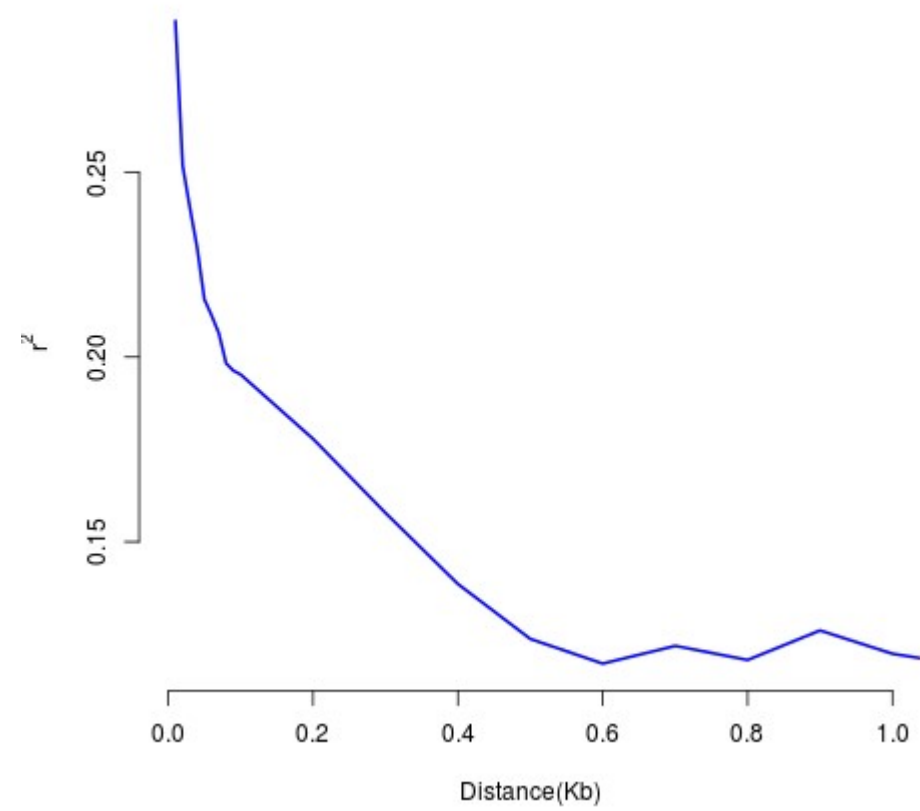

Supplement: Web_Material_uhae265 [file web_material_uhae265.zip › Aqbouch_etal_Figure_S7.pdf]
